# The Potential of Medicinal Plants and Natural Products in the Treatment of Burns and Sunburn—A Review

**DOI:** 10.3390/pharmaceutics15020633

**Published:** 2023-02-13

**Authors:** Weronika Skowrońska, Agnieszka Bazylko

**Affiliations:** Department of Pharmaceutical Biology, Faculty of Pharmacy, Medical University of Warsaw, Banacha 1, 02-097 Warsaw, Poland

**Keywords:** burn, sunburn, wound, plant extracts, *Albizia julibrissin*, *Aloe vera*, *Arnebia euchroma*, *Betula*, *Centella asiatica*, *Hippophaë rhamnoides*

## Abstract

Treating burns remains a challenge for modern medicine, especially in developing countries that cannot afford expensive, advanced therapies. This review article summarises clinical and animal model studies of botanical preparations and their mixtures in treating burn wounds and sunburn. Articles available in electronic databases such as PubMed, Scopus, Web of Science, Science Direct and Google Scholar, published in English in 2010–2022, were considered. In the described clinical trials, it was shown that some herbal preparations have better effectiveness in treating burn wounds, including shortening the healing time and reducing inflammation, than the conventional treatment used hitherto. These herbal preparations contained extracts from *Albizia julibrissin*, *Alkanna tinctoria*, *Aloe vera*, *Arnebia euchroma*, *Betula pendula* and *Betula pubescens*, *Centella asiatica*, *Hippophaë rhamnoides*, *Juglans regia*, *Lawsonia inermis*, and mixtures of *Matricaria chamomilla* and *Rosa canina*. Research on animal models shows that many extracts may potentially benefit the treatment of burn wounds and sunburn. Due to the diverse mechanism of action, antibacterial activity, the safety of use and cost-effectiveness, herbal preparations can compete with conventional treatment. The growing interest in alternative medicine and herbal medicine encourages further research. Not only single preparations but also their mixtures should be taken into account because the research conducted so far often suggests a synergistic effect of the ingredients.

## 1. Introduction

According to the World Health Organisation, burn injuries affect more than 11 million people yearly. More than 180,000 people die, and approximately 95% of deaths occur in low-income and developing countries. Due to high costs, modern therapies are available almost exclusively in developed countries. Poorly designed healthcare systems and low health expenditures per capita in low-income countries correlate with higher post-injury complications, which can lead to disability and death. Therefore, treating burn wounds remains challenging, particularly as cost-effective therapy [1,2].

Burns can be defined as tissue damage due to the action, in most cases, of high temperature, electricity, chemicals, and radiation. The classification of burns is based on their depth and size. There are four degrees based on the severity of the injury. Superficial burns, also known as first-degree burns, involve only the epidermis. They are characterised by redness and discomfort, sometimes pain, but usually do not require medical intervention. Second-degree burns can be divided into two subcategories, superficial partial-thickness burns and deep partial-thickness burns. A superficial partial-thickness burn covers the epidermis and part of the dermis. It is usually manifested by the appearance of blisters filled with serous fluid. It is painful and requires wound care and dressing but usually does not cause scarring. A deep partial-thickness burn covers the epidermis and dermis. It is deeper but usually less painful due to damage to the pain receptors. It leads to scarring and sometimes requires surgical intervention. Third-degree burns, or full-thickness burns, involve the entire skin. They are usually not painful because the nerve endings are damaged. Blood vessels and subcutaneous tissue are also damaged. Treatment is long and requires surgical removal of necrotic tissue, administration of antibiotics and usually a skin graft. In addition to the skin and subcutaneous tissue, fourth-degree burns involve muscles and bones. Charring is a characteristic picture. The changes are irreversible. They usually lead to amputation of the affected limb or death of the patients [3].

Burns are characterised by high susceptibility to bacterial infections. A damaged skin barrier, easy access to nutrients in the wound environment, damage to the wound vascularisation, lack of epithelialisation of the basal epidermal tissue, or systemic disorders leading to immunosuppression facilitate the entry of pathogens [4]. Therefore, topical antimicrobial agents are still very often used in treating burns. The most commonly used topical formulation and considered the standard therapy is 1% silver sulfadiazine (SSD) cream. However, in recent years, numerous disadvantages of this preparation have been described, including delaying healing or a cytotoxic effect on various host cells [5,6]. Therefore, new preparations containing, or no silver ions are introduced to the market, mainly in ready-to-use dressings. Studies show that burn wounds treated with new dressings heal faster and are easier to use. In addition, burns treated with the new dressings are less susceptible to secondary infection than treatment with 1% SSD cream [7,8].

Superficial and partial-thickness burns are the most common in pharmaceutical practice. The ideal dressing that we would like to recommend to a patient with a burn wound should, in addition to healing properties, have the following features: infection prevention, pain relief, moisture control, exudation removal, gas exchange, low skin adhesion, mechanical stable, reducing wound necrosis, cost-effective, non-toxic, biocompatible, and biodegradable [9]. However, individual regional needs or desires and the patient’s perspective and economic constraints must also be considered [10]. The answer to the requirements could be dressings or pharmaceutical preparations containing products of natural origin [11].

This review aimed to summarize knowledge on the use of plant preparations in the treatment of burn wounds. The paper includes articles from 2010 to 2022 describing clinical trials for single herbal preparations or their mixtures and animal studies of plants or plant extracts and their mixtures.

## 2. Materials and Methods

Electronic databases, including PubMed, Scopus, Web of Science, Science Direct, and Google Scholar, were searched for materials for this review. Articles published in English in the years 2010–2022 were selected. Search terms were “burn” and “sunburn” in the title or abstract, and “plant extract”, “plant”, and “herbal” in the abstract and full text.

Original papers describing the effect of individual plant preparations and mixtures on treating burns and sunburn were included. The paper describes clinical trials and studies on animal models in separate sections. In addition, the results for single formulations and mixtures are described separately. The descriptions or tables provide the scientific names of individual plant species or plants included in the mixture and the plant part used. Studies on single chemical compounds isolated from plants were not taken into account.

## 3. Results

### 3.1. Clinical Trials—Single Preparations

In 2010–2022, several clinical trials were conducted to check the effectiveness of single-plant preparations in treating burns. Studies have been conducted on the Persian silk tree (*Albizia julibrissin*), aloe (*Aloe vera*), pink Arnebia (*Arnebia euchroma*), silver birch (*Betula pendula*) and downy birch (*Betula pubescens*), tea plant (*Camelia sinensis*), gotu kola (*Centella asiatica*), sea buckthorn (*Hippophaë rhamnoides*), and common walnut (*Juglans regia*). Studies for individual species are described below. A summary of the results is provided in Supplementary Materials in Table S1.

#### 3.1.1. *Albizia julibrissin*

*Albizia julibrissin* from the Fabaceae family was originally found in South and East Asia, from Iran and Azerbaijan to China and Korea. In local folk medicine, the *Albizia* species has been used to treat melancholy, insomnia, fever, headaches and abdominal pain, diabetes, and rheumatism, but also to treat wounds, snake bites, haemorrhoids, abscesses, erysipelas, and leprosy. The main chemical components are triterpene saponins, but flavonoids, lignans, alkaloids, and phenolic glycosides are also present [12].

A prospective, randomised, double-blind clinical trial investigated the efficacy of a gel containing 5% (*w*/*w*) *Albizia julibrissin* extract in treating burns [13]. The extract was prepared by extracting the stem bark of *Albizia julibrissin* with 60% (*v*/*v*) ethanol. Forty patients with second and third-degree burns participated in the study. Patients were randomly assigned to two equal groups where 20 were treated with 5% *A. julibrissin* gel and the remaining 20 with 1% SSD cream. The wounds were washed once daily with a saline solution, and treatment was applied. The treatment was continued for 30 days. The study compared clinical parameters such as inflammation, pain, itching, erythema, oedema, purulent discharge, and skin discolouration. Before the beginning of the study, there were no significant differences in those parameters between patients from the two groups.

After 15 days of treatment, a statistically significant reduction in pain, inflammatory reaction and purulent discharge were observed in the group treated with 5% *A. julibrissin* gel compared to the group treated with 1% SSD cream. There were no significant differences between groups in itching, erythema, oedema, and skin discolouration. At the end of treatment, after 30 days, a reduction in inflammation and pain sensation was observed in the group treated with the 5% *A. julibrissin* gel. However, the other assessed parameters found no statistically significant differences between the groups. Treatment with 5% *A. julibrissin* gel shortened epithelialisation time in second and third-degree burns by 33.3 and 43.78%, respectively, compared to treatment with 1% SSD cream. The patients rated the colour, odour, and stability of 5% *A. julibrissin* gel worse than the 1% SSD cream.

#### 3.1.2. *Aloe vera*

*Aloe vera* (Asphodelaceae) has a long history of medicinal use, dating back to ancient cultures such as Chinese, Egyptian, and Indian. Over the years, several studies have been conducted on its pharmacological use. Its therapeutic activity includes, among others, antibacterial, antiviral, anticancer, antioxidant, anti-inflammatory, skin protective, wound healing, and regulating blood glucose and cholesterol levels. *Aloe vera* is known primarily for its beneficial effects on the skin, mainly its mucous gel filling the leaves, which is used in many cosmetic and pharmaceutical preparations [14].

The effectiveness of *Aloe vera* cream in treating second-degree burns was tested in a randomised and controlled clinical trial [15]. The study involved 30 patients with two thermal burns of similar size and depth on two different but similar body areas (such as hands or feet). The burn must have occurred within 24 h of treatment initiation and not affected more than 40% of the total body surface area. After cleaning the wound with saline solution, a base cream containing 0.5% pure spray-dried aloe powder (Zarband Phytopharmaceuticals, Teheran, Iran) was applied to one burn. A 1% SSD cream was applied to the second burn. Dressings were changed, and the cream was applied twice a day. Treatment was continued until complete epithelialisation of the burn.

Mean times to complete healing were 15.9 ± 2 and 18.73 ± 2.65 days for a burn treated with aloe cream and 1% SSD cream, respectively. The time to complete healing was statistically significantly shorter in the case of treatment with aloe cream. Additionally, the size of the wound treated with the aloe cream was significantly smaller after 10, 13, and 16 days. After days 3, 7, and 13, no microbial contamination was observed. This clinical study showed that aloe cream might be more effective in treating burns than 1% SSD cream, significantly reducing wound healing time and surface area.

Another randomised clinical trial compared the effectiveness of 98% *Aloe vera* gel with that of 1% SSD cream in treating second-degree burns [16]. The study involved 50 patients with second-degree heat burns that developed within 24 hours of starting treatment and did not exceed 25% of the total body surface area. The wound was washed with pyodine scrub and saline, and then aloe gel or 1% SSD cream was applied. Dressings were changed twice a day. Treatment was continued until the wound was completely healed and re-epithelialised.

The study compared the size and area of the wound as well as the patients’ subjective perception of pain. The mean wound epithelialisation time was 11 ± 4.18 and 24.24 ± 11.16 days for the aloe gel and 1% SSD cream treated groups, respectively, and was significantly shorter for those treated with aloe vera gel. There were no differences in the infection of the wounds of both groups. In the group treated with aloe vera gel, the time to complete pain relief was 21 days and was significantly shorter than in the group treated with 1% SSD cream, which was 26 days. The study showed that aloe vera gel significantly shortens the re-epithelialisation time, alleviates pain symptoms, and is more cost-effective.

#### 3.1.3. *Arnebia euchroma*

*Arnebia euchroma* from the Boraginaceae family occurs naturally in high mountain regions, mainly in the Himalayas and other regions of Asia and North Africa. It is a source of many promising chemical compounds from the group of naphthoquinones, mainly ester derivatives of shikonin, alkannin, and isohexenylnaphthazarin. Potential medicinal properties include wound-healing, antibacterial, antiviral, antifungal, anti-inflammatory, and anticancer effects [17].

The effectiveness of *Arnebia euchroma* ointment was tested in a prospective, randomised, single-blind clinical study compared to the effectiveness of 1% SSD cream [18]. To prepare the ointment, chopped dried roots of *A. euchroma* were heated in goat fat, cow butter, and glycerin at 95–100 °C for 30 min. The mixture was sterilised, filtered, and then Eucerin, methylparaben, and propylene paraben were added. The weight ratio of *A. euchroma* roots to primary materials was 10%.

The study involved 45 patients who suffered second-degree burns on two parts of their body, covering no more than 15% of their total body surface area. The burn was to appear within 24 h of starting treatment. The injured parts were randomly assigned to treatment with *A. euchroma* ointment (AEO) or 1% SSD cream, which was continued until complete wound healing. The wound was washed daily with saline solution, and appropriate treatment was applied. On days 1, 3, 5, 7, 10, 13, 15, 20, 25, and 30, the wound was measured, and photographs were taken prior to the application of the cream.

By the fifth day of treatment, no significant differences were observed in the size of the wound treated with AEO and 1% SSD cream. In the following days, the area of the wound treated with AEO was significantly smaller than that of the wound treated with SSD. The average wound healing time was 13.9 ± 5.3 days for AEO and 17.5 ± 6.9 days for 1% SSD cream. It was significantly lower for wounds treated with AEO. Physicians’ treatment preferences overwhelmingly favoured AEO from day 15 of treatment. The mean global assessment of wound appearance by the experienced nurse did not differ between the groups. Burning sensation and pain sensation were lower, while warming of the injury area was higher for the part treated with AEO than the part treated with 1% SSD cream. Patients’ satisfaction with AEO treatment was significantly higher than with 1% SSD cream treatment.

#### 3.1.4. *Betula pendula, Betula pubescens*

The leaves of *Betula pendula* and *Betula pubescens*, species of the Betulaceae family, rich in flavonoid compounds, have been used in traditional medicine as diuretics that increase urine flow, flush the urinary tract, and prevent the development of infections [19]. However, now researchers are interested in birch bark. Betulin, the main component of birch bark extract, was first described in 1788. Only in recent years has it gained importance as a pharmaceutical ingredient. Betulin has been found to have the ability to stabilize water-in-oil emulsions but not as a surfactant. In addition, it gels oils, thanks to which it creates thixotropic gels, the durability of which is higher at body temperature than at room temperature. After receiving excellent results in toxicology and pharmaceutical safety studies, the era of clinical trials began to clarify the indications where triterpene birch bark extract could be used [20].

Oleogel-S10 (tradename Episalvan^®^) is a sterile gel containing 10% birch bark extract and 90% sunflower oil. Triterpene birch extract, obtained from *Betula pendula*, *Betula pubescens* and mixtures of these species, is standardised for the content of betulin (72–88%), and it also contains, among others, betulinic acid, lupeol, oleanolic acid, and erythrodiol.

An open, blindly evaluated, randomised clinical trial was conducted to test the effectiveness of Oleogel-S10 in treating superficial partial-thickness burn wounds [21]. Based on the study set out below, the European Medicines Agency decided to approve Episalvan^®^ for treating second-degree burns. The obtained results were compared to treatment with octenidine hydrochloride gel (Octenilin^®^ wound gel, Schülke & Mayr GmbH, Germany). Patients with one superficial second-degree burn >80 cm^2^ and <25% of total body surface area or two comparable burns >40 cm^2^ and <12.5% of total body surface area were eligible for the study. After washing the wound with octenidine hydrochloride or polyhexanide, Oleogel-S10 was applied to one wound or half of a large burn, and Octenilin^®^ was applied to the other, both approx. 1 mm thick, and then covered with gauze. The wounds were washed and dressed every 2 days for 21 days. Finally, treatment parameters were compared in 57 patients.

The study showed that 35 patients out of 57 had differences in the time required for wound closure. Among them, 30 patients (85.7%) treated with Oleogel-S10 had earlier healing than 5 (14.3%) treated with Octenilin^®^. The statistical analysis showed the advantage of treatment with Oleogel-S10 over treatment with Octenilin^®^. The percentage of wound epithelialisation on each analysed day was significantly higher for burns treated with Oleogel-S10 compared to Octenilin^®^. Oleogel-S10 was rated “better” or “significantly better” than Octenilin^®^ by 73.7% of the investigators and 71.9% of the patients. Treatment with these preparations was considered comparable by 8.8% of the investigators and 12.3% of the patients. Only 1.8% of the investigators and none of the patients considered that treatment with Octenilin^®^ was “better” or “much better”. At the end of the treatment, the tolerability of treatment with Oleogel-S10 and Octenilin^®^ was assessed. Oleogel-S10 was rated as “better” or “much better” by 65.6% of the investigators and 65.6% of the patients. The treatment was considered comparable by 19.7% of the investigators and 18.0% of the patients. Only 1.6% of patients and none of the investigators considered the treatment with Octenilin^®^ “better” or “much better”.

After 3 months of continuation in which 43 patients participated, the treatment with Oleogel-S10 was superior to treatment with Octenilin^®^. After 12 months of follow-up with 25 patients, the same result was obtained.

#### 3.1.5. *Camellia sinensis*

*Camellia sinensis* from the Theaceae family is a rich source of compounds from the polyphenol group, mainly catechin, epicatechin, and their derivatives. It is known primarily for their strong antioxidant and anti-inflammatory properties. Many studies have proven its beneficial effects on the skin, including photoprotection, anti-ageing, and anti-cellulite. Moreover, they have been shown to improve the condition of hair and skin and its blood supply [22].

The effectiveness of a cream with 10% water extract from green tea leaves containing 85% catechins in the treatment of burns was tested in a clinical study compared to a 1% SSD cream [23]. The study involved 50 patients who developed second-degree thermal burns to less than 5% of their total body surface area within 24 hours of starting treatment. The patients were divided equally into two groups so that each group included patients with a similar body surface area affected by the injury. The wounds were cleaned with a saline solution, a cream with green tea extract (GT) or 1% SSD cream was applied directly to the burn, and a dressing was applied. The patients and those changing the dressing and making records did not know which cream had been applied. The wounds were cleaned, and cream was applied daily. Photographs of the burned area were taken just before applying the cream. Treatment and photographic documentation continued until complete epithelisation. Treatment progress was assessed daily using the Bates-Jensen assessment tool, which includes 13 parameters rated on a 5-point Likert scale. The parameters assessed included wound size, wound depth, wound edge, undermining, necrotic tissue type, necrotic type amount, exudate type, amount of exudate, surrounding skin colour, peripheral tissue induration, peripheral tissue oedema, granulation tissue, and epithelialisation.

There were no statistically significant differences in the assessment of treatment effectiveness in the GT cream and the 1% SSD cream groups, comparing the results between 2 and 14 days. Although in the 1% SSD cream group, only 2 patients had complete epithelialisation after 8 days, and as many as 7 patients in the GT cream group, finally, after 14 days of treatment, the number and time of complete epithelialisation did not differ. Moreover, slightly better (the difference was not statistically significant) patients from the GT cream group assessed the effect of treatment on peripheral oedema, the presence of granulation tissue and epithelialisation on days 8 to 12.

#### 3.1.6. *Centella asiatica*

*Centella asiatica* is a plant from the Apiaceae family derived from traditional Chinese medicine. The main chemical compounds responsible for its action are terpenoids, mainly asiaticoside, asiatic acid, madecassoside, and madecassic acid. The potential therapeutic effect is mainly related to the influence on the nuclear factor kappa-light-chain-enchancer of activated B cells (NF-κB), migoten-activated protein kinase (MAPK), glycogen synthase kinase 3β (GSK-3β), phosphoinositide 3-kinases/protein kinase B (PI3K/AKT), transforming growth factor β1/Smad (TGF-β1/Smad), and Janus kinases/signal transducer and activator of transcription proteins (JAK/STAT) pathways. Clinical studies have proven, among others, the effect on improving cognitive functions, alleviating anxiety, supporting wound healing or having a beneficial effect on skin care [24].

In a prospective randomised clinical trial, Centiderm^®^ ointment and 1% SSD cream were compared in parallel to treat second-degree burns [25]. Centiderm^®^ ointment contains the butanolic fraction of ethanolic extract (approx. 3%) from *Centella asiatica* leaves and is made with Vaseline and glycerine. Patients with second-degree burns on a limb that covered no more than 10% of the total body surface and occurred within 48 hours of the start of treatment were eligible for the study. Finally, 60 patients randomly assigned to two equal groups were analysed. Centiderm^®^ ointment or 1% SSD cream was applied to the burn once a day until complete healing. On days 0, 3, 7 and 14 of the study, objective (pliability, vascularity, pigmentation, heigh, visual acuity score (VAS) and scoring according to Vancouver Scar Scale (VSS)) and subjective (dryness, itching and irritation) indices were assessed. In addition, the time needed for re-epithelialisation and complete healing was assessed.

Statistically, significantly more favourable effects of Centiderm^®^ ointment were observed compared to 1% SSD cream from the 3rd day of treatment. Pliability, height, vascularity, VAS and VSS were rated significantly better in the group treated with Centiderm^®^ ointment. The exception was pigmentation, for which no differences in the assessment were observed on the seventh day. However, on the 3rd and 14th day, it was assessed more favourably in the Centiderm^®^ group. Also, according to the patients’ subjective assessment of dryness, irritation and itching, the use of Centiderm^®^ ointment was more effective and prevailed over 1% SSD cream. The mean time to re-epithelialisation of 13.7 ± 1.48 and 20.67 ± 2.02 days for the Centiderm^®^ group and the 1% SSD cream group, respectively, was significantly shorter for the Centiderm^®^ group. On average, complete wound healing was 14.67 ± 1.78 days in Centiderm^®^ versus 21.53 ± 1.65 days in 1% SSD cream, which was a statistically significant difference.

#### 3.1.7. *Hippophaë rhamnoides*

*Hippophaë rhamnoides* is a plant of the Elaeagnaceae family that has been cultivated and harvested for its nutritional and medicinal properties since ancient times. It is used mainly due to its anti-diabetic, anti-obesity, and cardiovascular-improving properties. When used topically on the skin, its protective effect against solar radiation is emphasised [26,27].

In a randomised, triple-blind clinical trial, the effectiveness of sea buckthorn cream and 1% SSD cream in treating burns was investigated and compared [28]. The study involved 30 patients with second-degree thermal burns that affected no more than 10% of their total body surface area and occurred within 6 hours of arrival at the hospital. Patients were randomly assigned to two equal groups. The burns of the first group were treated with 1% SSD cream, and the burns of the second group with sea buckthorn cream. Sea buckthorn cream in 100 g contained 40 g of active ingredients from fresh fruits of *Hippophaë rhamnoides*. Once a day, after washing the wound with sterile normal saline, the cream was applied to a thickness of 3 mm. The study was completed by 27 patients in the first group and 28 in the second group.

The average wound healing time in the group treated with sea buckthorn cream was 6.7 ± 2.1 days and was statistically significantly lower than in the group treated with 1% SSD cream, which was 11.2 ± 2.3 days.

#### 3.1.8. *Juglans regia*

Walnuts (*Juglans regia*) from the Juglandaceae family are valued primarily in Asia and Europe for their nutritional properties. They are a rich source of unsaturated fatty acids, proteins, vitamins, and minerals. Phytosterols, flavonoids, and polyphenols are also present. Due to their antioxidant, anti-inflammatory, and antibacterial properties, walnuts have been used in folk medicine to treat acne and eczema [29].

The Department of Burns and Plastic Surgery of the General Hospital of Ningxia Medical University (Ningxia, China) developed a walnut-based ointment decades ago that was successfully used to treat non-healing burn wounds. In a retrospective evaluation of cases, it was decided to compare the effectiveness of treatment with walnut ointment, conventional treatment, and surgery [29]. The study enrolled 411 patients with burn wounds covering 0.1 to 7% of the total body surface area, which were classified as non-healing. In the experimental group (49 patients), the burns were covered with a layer of 1–2 mm thick walnut ointment, prepared by crushing the nuts, heating them for 30 min, and grinding them into a paste. In 165 patients receiving conventional treatment, wounds were treated with an antimicrobial agent and recombinant human epidermal growth factor (rhEGF), 88 patients were treated with silver ion dressing + rhEGF, 42 patients were treated with Polymyxin B + rhEGF, and 35 patients were treated with Gentamicin + rhEGF. Patients qualified for the surgical group (197 people) received wound debridement and skin autograft.

The successful cure was reported for 76.60% of cases in the experimental group, 75.13% in the surgical group, and only 9.70% in the conventional treatment group. Treatment with walnut ointment was statistically as effective as surgery, and both treatments were superior to conventional treatment. The time necessary for complete wound closure was 19.87 ± 9.10 days for the experimental group and 22.71 ± 11.77 days for the surgical group, and it was significantly shorter than in the conventional treatment group, where it was 36.67 ± 10.18 days.

### 3.2. Clinical Trials—Mixtures of Natural Products

From 2010–2022, six clinical trials were also conducted to test the effectiveness of treating burn wounds with mixtures of plant-origin preparations. They are described below and summarised in Table S2 in Supplementary Materials.

#### 3.2.1. *Alkanna tinctoria*, Olive Oil, and Beeswax

The effectiveness of a mixture of *Alkanna tinctoria*, beeswax and olive oil in the treatment of burn wounds was tested in a clinical study [30]. The mixture was prepared by adding 30 g of beeswax to 1000 mL of medical olive oil brought to the boiling point (200–210 °C), and then, after its complete melting, 50 g of *Alkanna tinctoria* (the part of the plant used was not specified) was added and heated for 5 min. Afterwards, the mixture was filtered and dispensed into bottles which were then sterilised. Dressings were prepared immediately before application by saturating a sterile sponge with the mixture.

The study ultimately compared the results of 64 patients (33 in the control group and 31 in the experimental group) with thermal burns caused, in most cases, by boiling liquids within 24 h of admission to the hospital. There were no statistically significant differences in injury characteristics at the start of the study. Dressings were changed every two days using aseptic techniques under sterile conditions. The wound was washed with normal saline and 0.1% chlorhexidine digluconate, and dressings were applied. In the experimental group, it was a dressing saturated with a previously prepared mixture, while in the control group, a standard dressing used in this hospital for burns with nitrofurazone and rifamycin was used.

The time to start re-epithelialisation was 3.0 ± 0.85 days in the experimental group and was significantly shorter than in the control group, which was 6.79 ± 1.77 days. The average pain experienced by patients was significantly lower in the experimental group (8.12 ± 1.38 points) than in the control group (9.39 ± 1.05 points). In addition, the use of treatment with a natural mixture significantly reduced the duration of hospitalisation. In the control group, it was 14.42 ± 7.79 days, and in the experimental group, it was only 8.22 ± 3.05 days.

#### 3.2.2. *Aloe vera* and *Centella asiatica*

A randomised, prospective clinical trial compared the effectiveness of treatment of second-degree burns with a dressing containing *Aloe vera* and *Centella asiatica* and a commercial Bactigras^®^ (Smith & Nephew, Hull, UK) dressing [31]. Thirty-five patients with second-degree burns covering at least 20% of their total body surface area were randomly divided into two groups. The experimental group was treated with a dressing impregnated with lipocolloids, 5% of *Centella asiatica* cream (Cosmelene^®^), 2.5% spray-dried powder of *Aloe vera* gel, and the standard group with a dressing impregnated with soft paraffin and 0.5% chlorhexidine acetate. Dressings were changed every 3 days until complete wound healing. Each time, the wound surface was measured, and the patient’s pain was assessed 30 minutes after applying a new dressing.

The time to complete healing in the group treated with dressings with herbal extracts was 18.53 ± 1.66 days and was significantly shorter than in the group treated with a standard dressing (20.06 ± 2.51 days). Moreover, the hospital patients’ stay was significantly shortened, from 22.78 ± 2.58 days in the standard group to 21.12 ± 1.83 days in the experimental group. The percentage of epithelialisation was significantly higher in the experimental group from day 15, and the mean pain the patient experienced was lower than the standard group.

There was one *Pseudomonas aeruginosa* infection in the experimental group on day 7. Therefore, the patient received standard treatment and dropped out of the clinical trial. No alarming symptoms or side effects of treatment with *Aloe vera* and *Centella asiatica*-impregnated dressings were observed.

#### 3.2.3. *Aloe vera*, *Lavandula stoechas*, and *Pelargonium roseum*

A randomised, double-blind clinical trial investigated the effectiveness of a herbal mixture containing *Aloe vera* gel, *Lavandula stoechas*, and *Pelargonium roseum* essential oils in treating burns [32]. The exact composition of the preparation has not been provided. The results were compared with standard treatment with 1% SSD cream. The study enrolled 120 patients with second-degree burns that developed within 48 h of treatment and covered less than 5% of the total body surface area. The treatment consisted of daily dressing changes preceded by cleaning the wound with an antimicrobial solution and applying a cream (5 g per 10 cm^2^ of the injured area). Patients were assessed for pain intensity, skin dryness, and infection.

The study was completed by 111 patients. There were 56 people in the experimental group and 55 in the standard group. There were no statistically significant differences in the occurrence of dry skin between the groups at any time. Both groups had a reduction in pain compared to the first day. In the experimental group, pain intensity was significantly lower on the 7th day than in the standard group. There was only one case of infection in the experimental group that resolved during continued treatment.

#### 3.2.4. *Azadirachta indica* Oil and *Hypericum perforatum* Oil

In a retrospective, non-controlled study, the effectiveness of a plant preparation in a spray (1 Primary Wound Dressing^®^; Phytoceuticals AG, Zurich, Switzerland) in treating burn wounds was checked [33]. The product contains hypericum oil (*Hypericum perforatum*) and neem oil (*Azadirachta indica*), which, when applied directly to the wound, creates a mist that provides an appropriate wound healing environment and does not adhere to the wound. The review was performed on 9 paediatric patients with 18 wounds in total. Granulation tissue formation, epithelisation, wound surface, pain sensation, and time to healing were assessed.

After a few days of using the preparation, granulation tissue formation and epithelisation were induced. The average time needed for wound healing was 16.6 ± 4.69 days. In six patients, a strong relief of pain was observed (from about 7–8 out of 10 points to 0) in the first week of using the preparation. In the remaining patients, the pain subsided within the second or third week. No adverse effects of the therapy, such as an allergic reaction or infection, were observed.

#### 3.2.5. *Lawsonia inermis* and Beeswax

Originating from Iran, the herbal ointment Fundermol, which contains *Lawsonia inermis* and beeswax, has been used to treat severe burns. The exact composition of the preparation has not been provided. Its effectiveness was tested in a clinical study for treating second-degree burns compared to 1% SSD cream [34]. The study involved 50 patients with burns covering 1 to 10% of the total body surface, which resulted from contact with a heater or hot liquid within 6 hours of arrival at the clinic. Patients were randomly assigned to two equal groups, treated once daily with Fundermol ointment or 1% SSD cream, respectively.

The average wound healing time in the group treated with Fundermol ointment was 4.4 ± 1.87 days and was significantly shorter than in the group treated with 1% SSD cream, which was 5.9 ± 2.20 days.

#### 3.2.6. *Matricaria chamomilla*, *Rosa canina* and Beeswax

Adibderm^®^ ointment is a herbal preparation which includes chamomile and rose extracts, ascorbic acid, beeswax, and oleic and linoleic acids. The formulation activity was tested in a randomised clinical trial involving 60 patients with second-degree burns covering 1–10% of the total body surface area, which occurred within 2 h of admission to the emergency room [35]. Patients were randomly assigned to two groups where patients applied herbal ointment and 1% SSD cream every six hours. The average wound healing time was 7.53 ± 2.28 days in the herbal ointment group and 11.83 ± 2.32 days in the 1% SSD cream group. The difference was statistically significant.

Patients’ satisfaction with herbal treatment was significantly higher than conventionally treated patients. In the group treated with Adibderm^®^ ointment, there were no cases of infection, but 7 patients developed irritation, while in the group treated with 1% SSD cream, there was one case of infection and no irritation.

### 3.3. In Vivo Studies on Animal Models of Burn

In order to better understand the physiological and pathophysiological mechanisms associated with burn injury, in vivo models are used in which animals, mainly mice, rats, guinea pigs, rabbits, hamsters, and sometimes pigs, are used. None of these could be considered better than the others. Rather, they should be considered complementary and show basic mechanisms that may not always reflect the pathology of a burn in humans. Mice and rats are the most commonly used models as they are cheap and have a high reproductive rate. However, there are many differences compared to humans, including their size, anatomy, and metabolic characteristics. It has been shown that, compared to mice or rats, guinea pig skin is anatomically and physiologically more similar to human skin. Due to, among other things, the thickness of the epidermis, the guinea pig burn model better reflects the thermal skin of a human wound [36]. The model closest to humans is pigs. However, they are very expensive to maintain, require increased care and carry a higher risk of infections [37].

Burns are usually formed on the previously hairless backs of animals and cover 5 to 30% of the total body surface area [36]. Animals are obligatorily anaesthetised with pharmacological agents such as ketamine, xylazine, diazepam, midazolam, thiopental and others, or mixtures thereof, and a burn is induced [38]. The most important animal models of burn wound formation include the gas flame burn model, burning ethanol bath burn model, pre-heated single metal plate/bar burn models, boiling or hot water burn models, and pre-heated double brass blocks burn model [36]. The assessment of burn healing is based on the time of epithelisation, wound closure, and histopathological analysis. Biochemical parameters can also be assessed, including the activity of superoxide dismutase, catalase, glutathione S-transferase, hydroxyproline content, or total protein content. Occasionally, the degree of hair regrowth in the injured area can be assessed [38].

In vivo studies in animal models of the effectiveness of preparations of plant origin in treating burn wounds in the single form are presented in Table 1, while mixtures of preparations are presented in Table 2.

## 4. Discussion

Burns significantly affect the quality of life of patients. Although they are common worldwide, they are a significant problem in developing countries, mainly because health care in every country cannot provide access to the latest, most effective therapies. Treatment, for example using only creams with antibacterial agents such as 1% silver sulfadiazine cream, can prolong healing time, lead to complications, and increase antibiotic resistance. Therefore, the search for new, safe, effective, and cost-effective preparations supporting burn healing is ongoing [2,107]. Burn injury affects the patient’s physical health, quality of life, and mental health. Therefore, the challenge is not only the wound treatment itself, which should be effective, but also possible in the patient and long-term care and rehabilitation in more serious cases [3].

Burn is accompanied by an inflammatory and immune reaction, metabolic changes, and distributive shock, especially in the case of severe burns. These symptoms can be difficult to manage, leading to multiple organ failure. An important factor in assessing the treatment needs of a burn injury patient is the wound’s depth [108]. In severe burns, highly deregulated inflammation develops, characterised by the release of inflammatory cytokines, chemokines, and acute phase proteins. During this violent phase, the immune system is stimulated and reacts inadequately to stimuli. Therefore, the anti-inflammatory effect of preparations on burn wounds is highly recommended [109,110]. The inflammatory phase is followed by the proliferation phase, during which primarily keratinocytes and fibroblasts are stimulated to rebuild tissue and vessels. In the final phase of healing, the wound remodels. Collagen and elastin are deposited, and fibroblasts are transformed into myofibroblasts. In the case of incorrect and fibrous location of collagen fibres and imbalance in the re-epithelialisation process, scar formation may occur [3,111].

Although several therapeutic activities have been demonstrated for plant-derived products, which are also beneficial in the treatment of burn wounds and sunburn, their preparation is associated with certain limitations. The chemical composition of plants and extracts made from them may be subject to certain deviations due to many factors. The place and time of harvesting, the level of insolation, the geographical altitude, the method of drying and fragmentation are some of the factors that have a significant impact on the chemical composition of the plant material [112,113]. Then, the method of extraction, selection of solvents, the ratio of plant material to solvent, time, and temperature of extraction, as well as the method of its drying and purification affect the composition of the extract. Therefore, it is necessary to introduce certain procedures for standardisation and assessment of the chemical composition when working with plant material [114].

**Figure 1:**
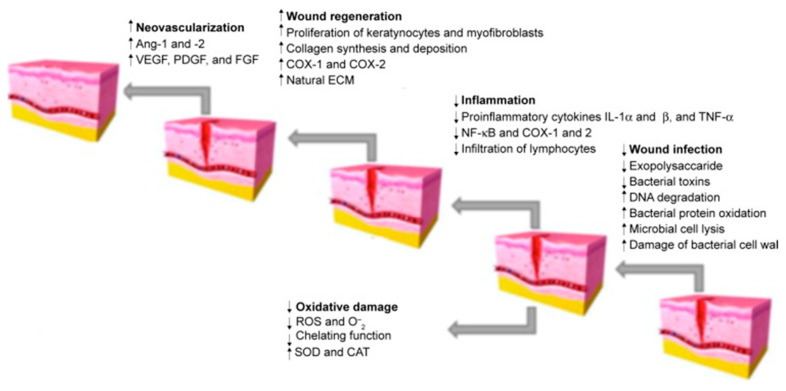
Stages of wound healing. Adapted from Hajialyani, M.; Tewari, D.; Sobarzo-Sánchez, E.; Nabavi, S.M.; Farzaei, M.H.; Abdollahi, M. Natural Product-Based Nanomedicines for Wound Healing Purposes: Therapeutic Targets and Drug Delivery Systems [119].

The next step that has a significant impact on the activity is the appropriate formulation of the finished product that will achieve the intended effect in the biological system. Most of the formulations presented in this review are traditional products, such as ointments and creams, containing previously prepared extracts. However, in recent decades, the approach to wound care and dressing has completely changed. Patients more and more often use ready-made dressings, e.g., polymer or hydrogel, which contain incorporated active substances released in a controlled manner. This method of the formulation is also increasingly used for herbal products [115,116]. Another new way of preparing natural preparations is nanoformulation. The development of methods using nanotechnology can support the effectiveness of herbal products. In the treatment of dermatological diseases, various novel drug delivery systems can be used, which contain compounds or extracts of interest in the form of, among others, nanoparticles, liposomes, nanoparticle polymers, nanohydrogels, and nanofibres. Some studies have confirmed that they may be more effective than conventional systems [117,118]. Plant extracts prepared in the form of nanoparticles are characterised by higher bioavailability and penetration of biological membranes, as well as a controlled release at the target site [119]. The role of natural products in different stages of wound healing is presented in Figure 1. It can be assumed that soon, natural compounds used in the form of nanopreparations will be the basis for the creation of new pharmaceutical drugs. However, it is necessary to conduct clinical trials that would verify these theses. In addition, studies are needed to compare the effectiveness compared to standard treatment, as well as to check whether isolated chemical compounds or extracts work better in specific clinical situations.

**Figure 2:**
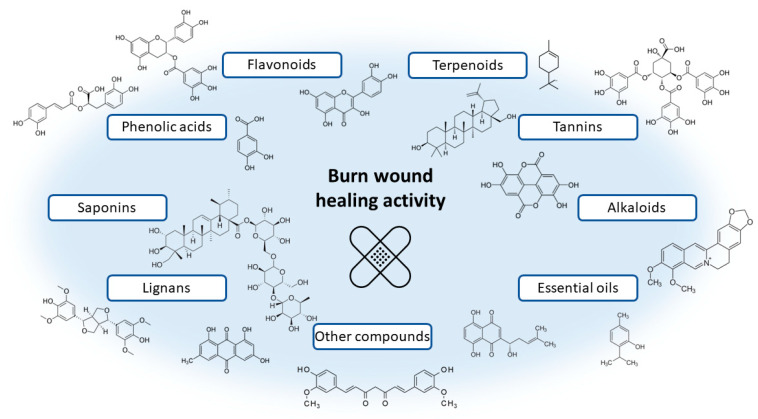
Examples of chemical structures of naturally occurring compounds involved in the wound healing process.

This review focuses on plant extracts and their mixtures that have been tested in animal models and clinical trials. Extracts, as mixtures of chemical compounds, can show different directions of action affecting the various stages of wound healing (Figure 1). In the inflammation phase, compounds with antibacterial, antioxidant, and anti-inflammatory effects will be particularly important. These include mainly polyphenols and flavonoid compounds, as well as essential oils [120,121]. They may have an antibacterial effect, as well as modulate the inflammatory response by regulating the secretion of cytokines and chemokines, such as interleukin 1β or interleukin 8, or tumour necrosis factor α (TNF-α). Studies on the effect of extracts and chemical compounds on the inflammatory phase are quite popular and there are more and more publications describing this issue [122]. However, less is known about the impact of individual chemical compounds on the proliferation and remodelling phase of wound healing. It is assumed that compounds from the groups of alkaloids, tannins, flavonoids and terpenes are of the greatest importance [11,120]. Important for the proliferative phase will be compounds that affect the formation of the extracellular matrix and stimulate re-epithelialisation, angiogenesis, or the formation of granulation tissue through, among others, stimulating cell proliferation and increasing the expression of proteins such as transforming growth factor β (TGF-β) or vascular endothelial growth factor (VEGF). Finally, in the remodelling phase, chemicals that affect, for example, stimulation of collagen deposition and elastin fibres [11,123] will be necessary. Thanks to the diverse mechanisms of action, potential antibacterial effect and safety of use, natural preparations compete with conventional treatment, all the more that the public’s interest in traditional medicine and herbal medicine is growing [124]. The chemical structures of the compounds involved in the wound-healing process are presented in Figure 2.

The results of clinical trials show that herbal preparations may work as well or better than conventional drugs. Preparations containing extracts from *Albizia julibrissin*, *Aloe vera*, *Arnebia euchroma*, *Centella asiatica*, *Hippophaë rhamnoides*, *Lawsonia inermis*, and the mixture of *Matricaria chamomilla* and *Rosa canina* work significantly better than 1% silver sulfadiazine cream, which was considered the “gold standard” of burn treatment until recently. Preparations containing extracts from *Betula*, *Juglans regia*, *Alkanna tinctoria*, *Aloe vera*, and *Centella asiatica* have shown in clinical trials an advantage over other conventional drugs, mainly containing antimicrobial agents. Considering the above results, the possibility of using herbal preparations, such as the marketed drug Episalvan^®^ containing birch bark extract, should be considered to be on par with standard drugs. Additionally,, in many studies of herbal formulations in animal models listed in Table 1 and Table 2, their superiority over treatment with antimicrobial agents, mainly 1% silver sulfadiazine cream, was demonstrated. Therefore, it is important to continue searching for new plant substances that potentially benefit burn wound healing. First, it is necessary to conduct clinical trials for promising formulations. Such research can contribute to introducing new drugs with documented beneficial effects that are safer and more affordable and would be available to all patients worldwide. In addition, considering the results in Table 2 and Table S2 (Supplementary Materials), the benefits of combining several plant substances in preparations should be considered. Many studies suggest a synergistic effect and may be more beneficial in treating burn wounds.

Scientific, clinical, and animal studies should be well-planned. In some of the studies presented above, a positive control group treated with a standard preparation with known therapeutic activity was not designed. That makes it difficult to interpret the results and unambiguously assess the tested preparation’s effectiveness. In addition, the tests performed should be described in detail. The lack of information about the research model, such as the conditions of creating a burn wound, make it impossible to reproduce the study in another centre, for example, to compare the results. In the case of market preparations, some authors did not provide information on their exact composition and manufacturer. Any deficiencies were noted in the individual studies in the tables. Appropriate interpretation of the obtained results and a correctly performed statistical analysis is essential. Some of the studies lacked statistical comparisons between the individual study groups, which made it difficult to analyse the results.

Researchers studying preparations of natural origin should pay particular attention to the accurate representation of the plant material under study. The chemical composition of plants changes depending on many factors, such as geographical altitude or insolation in the natural site [113]. Changes in chemical composition determine changes in therapeutic activity, which is why it is important to standardize the obtained extracts. Not all authors paid sufficient attention to describe and characterize the tested products adequately. Some studies lacked basic information, such as the final concentration or the amount of preparation applied to the burn.

What is more, sometimes the authors did not provide the method of preparation and did not even specify the part of the plant used. Such errors and oversights lead to a loss of credibility and trust among other scientists and doctors [125]. In conclusion, new research is needed for promising herbal products, but they should be carried out following all guidelines for this type of research.

## 5. Conclusions

Due to the diverse mechanism of action, antibacterial activity, and safety, herbal preparations compete with conventional treatment in treating burns and sunburn. The growing interest in alternative therapies and herbal medicine is also generating demand for such products. However, there is still a lack of clinical trials that would check the effectiveness of preparations showing beneficial effects on animal models of burns. Creating an ideal dressing for burn wounds that could replace the common use of antibacterial agents is a challenge for modern medicine, and the research presented in this review suggests that formulations based on herbal products are a strong competition for synthetic compounds.

## Figures and Tables

**Table 1:** In vivo studies on animal models—single preparations.

Plant Material	Animal Model	Burn Wound	Treatment Schedule and Study Groups *	Results	Ref.
*Aegialitis rotundifolia* leaves ethanolic extract	Wistar albino rats, male	Chemical burn—a few drops of concentrated hydrochloric acid Thermal burn—metal rod heated over the open flame for 30 s	Once a day for 18 days E: 2.5% and 5% (*w*/*w*) in the simple ointment S: 1% SSD cream C: simple ointment	The experimental and standard groups significantly increased the percentage of wound closure and decreased epithelisation time after chemical and thermal burns compared to the control group.	[39]
*Achillea millefolium* aerial parts ethanolic extract	New Zealand white rabbits, male	Third-degree thermal burn—heated metal plate (170 °C) applied for 10 s	Once a day for 21 days E: 5 mL of extract C: 5 mL of normal saline	From the 7th day of treatment, the wound area in the experimental group was significantly smaller than in the control group. After histopathological analysis in the experimental group, complete filling with granulation tissue, an increased amount of collagen fibres and a decrease in the number of inflammatory cells were observed, while in the control group, only fresh granulation tissue was observed. The number of isolated microorganisms decreased in the experimental group.	[40]
*Achyranthes aspera* leaves methanolic extract	Albino rats, either sex	Third-degree thermal burn—metal rod heated to 85 °C, pressed for 20 s	Twice a day for 7 days E: 5.0% (*w*/*w*) of extract in soft white petroleum S: Himax^®^ (traditional Ayurvedic ointment) C: soft white petroleum	On the 8th day, the wound area of the experimental group was significantly smaller than that of the control and standard groups. From the biochemical parameters, a higher content of protein, vitamin C, glutathione, catalase, superoxide dismutase, and hydroxyproline were noted in the experimental group than in the control and standard group. The concentration of matrix metalloproteinase 9 and matrix metalloproteinase 2 was higher in the experimental than in the control group. Moreover, there were more collagen fibres, and the proliferation of fibroblasts increased.	[41]
*Actinida deliciosa* fruits freshly sliced	Wistar albino rats, male	Second-degree thermal burn—hot plate warmed up to 110 °C, placed for 10 s	Once a day for 21 days E: sliced fresh kiwifruit (3 mm thick) S: 1% SSD cream C: Vaseline	The wound area in the experimental group was significantly smaller, and complete wound closure was observed faster than in the standard and control groups. In the macroscopic evaluation, in the experimental group, compared to other groups, an increase in hyperaemia was observed in the first days, and after 11 days, a significant decrease, moreover after 5 days, a reduction in oedema, as well as earlier epithelisation. Histopathological analysis showed significantly less inflammation and a higher score of vascularisation than the other groups, as well as a score of granulation similar to the standard group. Fewer bacteria were isolated from the wound of the experimental group. It has been shown that applying kiwi to a wound results in its enzymatic debridement.	[42]
*Actinida deliciosa* fruits freshly cut kiwifruit mixed with kiwi juice	Sprague-Dawley rats, male	Third-degree thermal burn—copper stamp, kept at 90 °C for 15 min	Once a day for 30 days E: freshly cut kiwifruit mixed with kiwi juice C: neutral ointment	After 20 days, the eschar separation was significantly accelerated in the experimental group. In addition, a significantly reduced wound surface area and better wound closure were observed than in the control group. No differences were observed in the microscopic assessment of the degree of vascularisation, collagen precipitation and acute and chronic inflammation level.	[43]
*Allium cepa* bulbs poultice	Holtzman albino rats, male	Second-degree thermal burn—hot 60-watt bulb, applied 3 times for 20 s	Once a day for 21 days E: 4 g of poultice S: 1% SSD cream C: w/o treatment	The wound area after 21 days in the experimental and control groups was similar and significantly smaller than in the control group. In the experimental group, the histopathological examination showed the presence of skin composed of a reticular stratum of fibroblasts, collagen, and several blood vessels. In contrast, only fibroblasts and collagen were present in the standard group; in the control group, a hyperaemic chorion was.	[44]
*Aloe vera* fresh leaves dry powder of leaf gel	Wistar albino rats, male	Second-degree thermal burn—hot water (90 °C), applied for 6 s	Twice a day for 25 days E: 0.5% of *Aloe* gel powder in base cream S: 1% SSD cream C: base cream N: w/o treatment	After 25 days, the mean wound size was 0.78 ± 1.3, 4.1 ± 3.6, 4 ± 2.3, and 5.5 ± 3 for the experimental, standard, control, and negative control groups, respectively, and was significantly lowest in the experimental group. The biopsy showed that epidermal re-epithelialisation and skin fibrosis were observed in the experimental group. Inflammation and granulation tissue were minimal, and no bacteria were found. Wound healing was significant in this group compared to the standard group. In the control groups, healing was minimal or negligible, and bacteria were present in the wounds.	[45]
*Aloe vera* fresh leaves dry powder of leaf juice	Wistar albino rats, female	Second-degree thermal burn—a piece of the aluminium heated to 100 °C, applied for 15 s	Once a day for 30 days E: 1 mL of 2% *Aloe* gel NC: w/o treatment	After 24 days, significant differences in the degree of wound closure and epithelialisation were observed between the groups. In the experimental group, wound closure was 95.64 ± 1.99%, and the time of complete epithelisation was 27 days, while in the control group, it was 80.15 ± 2.80% and 32.5 days, respectively. Histopathological analysis showed that after 18 days of treatment in the experimental group, the number of inflammatory cells was significantly lower, and the degree of epithelialisation and neo-angiogenesis was significantly higher compared to the control group.	[46]
*Aloe vera* leaves 30% (*v*/*v*) methanolic extract	Wistar albino rats, male	Second-degree thermal burn—metal plate heated in boiling water for 5 min, applied for 10 s	Twice a day for 21 days E: 0.5, 1, 1.5 or 2% of extract in Eucerin S: 1% SSD cream C: Eucerin N: w/o treatment	The degree of wound closure in the experimental groups was higher than in the control groups. After 21 days, the wound area of the group treated with 1.5 and 2% extract was smaller than in the standard group. Histopathological analysis showed better wound healing parameters for the experimental and standard groups than in the control group, such as the number of hair follicles, sebaceous glands, fibroblasts, macrophages, neutrophils, blood vessels, and the thickness of the epidermal layer.	[47]
*Anredera cordifolia* leaves ethanolic extract	White rats (*Rattus norvegicus*), male	Thermal burn—iron plate soaking in boiling water for 5 min, applied for 30 s	Three times a day for 14 days E: 2.5, 5 or 7.5% of extract in Vaseline S: 1% SSD cream	Statistically, the best results were obtained in the experimental group treated with 5% of the extract. The highest degree of collagen deposition, the lowest infiltration of polymorphonuclear cells, mild angiogenesis, and moderate fibrosis were observed there. In the standard group, the degree of healing was the lowest.	[48]
*Arnebia euchroma* whole plant 50% (*v*/*v*) ethanolic extract	Sprague-Dawley rats, female	Third-degree thermal burn—iron plate heated to 100 °C, applied for 40 s	Once a day for 17 days E: 10 or 20% of extract in a carboxymethylcellulose (CMC) gel C: CMC gel N: w/o treatment	The degree of wound closure after 18 days was significantly higher in the group treated with 20% of the extract. However, the histopathological analysis showed the highest content of fibroblasts, collagen, and blood vessels in both experimental groups.	[49]
*Arnebia euchroma* leaves and root unspecified extract	Wistar albino rats, female	Second-degree thermal burn—aluminium plate heated to 60 °C, applied for 5 s	Once a day for 28 days E: 10 or 20% (*v*/*v*) of extract in vehicle gel S: 1% SSD cream N: w/o treatment	Compared to the control group, the treatment groups showed an increase in the amount of granulation tissue, degree of epithelisation, reduction of the wound area, fibroblast proliferation, volume of collagen fibres, and length and diameter of blood vessels. Wound closure was the fastest in the group treated with 10% extract, and fibroblast proliferation was the highest in the group treated with 20%.	[17]
*Azadirachta indica* leaves 90% (*v*/*v*) ethanolic extract	Wistar albino rats, male	Second-degree thermal burn—hot wax (80 °C), applied until solidified	Once a day (0.5 g per wound) for 15 days E: 1% of extract in base gel S: 1% SSD cream C: base gel	The average wound closure after 15 days in the experimental group was 81.25 ± 0.7822%, and in the standard group, 90.43 ± 0.7691%, which were higher values than in the control group (68.58 ± 0.7791%). The average re-epithelialisation time was 28.77 ± 1.22 days in the experimental group and 25.20 ± 2.10 days in the standard group, while in the control group, it was significantly longer–38.36 ± 1.77 days.	[50]
*Bauhinia purpurea* leaves methanolic extract	Sprague-Dawley rats	Second-degree thermal burn—hot molten wax (80 °C), applied until solidified	Once a day for 22 days E: 2.5 or 5% of extract in a simple ointment S: 5% of *Aloe vera* extract C: simple ointment	The wound epithelialisation period was 15.83 ± 0.30, 14.33 ± 0.49, 12.16 ± 0.40 and 13.13 ± 0.40 days for the control, treated with 2.5% extract, treated with 5% extract and standard groups, respectively. The period was statistically significantly shorter for the group treated with 5% extract and the standard group compared to the control.	[51]
*Bauhinia purpurea* leaves chloroform extract	Sprague-Dawley rats	Second-degree thermal burn—hot molten wax (80 °C), applied until solidified	Once a day for 22 days E: 2.5 or 5% of extract in Carbopol base S: 5% of *Aloe vera* extract C: Carbopol base	The period of wound epithelialisation in the group treated with 5% of the extract and the standard group was 14.50 ± 0.42 and 13.13 ± 0.40 days, respectively, and was statistically significantly shorter than in the control group and the group treated with 2.5% extract, in which it was 16.50 ± 0.50 and 16.33 ± 0.49 days, respectively.	[51]
*Brassica oleracea* leaves aqueous extract	Sprague-Dawley rats, female	Second-degree thermal burn—hot metal stamp (80 °C), applied for 10 s	Once a day for 28 days E: 1 g of extract per 1 mL base cream (70% sorbitol in glycerin) S: 1% SSD cream C: base cream NC: w/o treatment	Compared to other groups, the experimental group showed a decrease in the fibrinoleukocytic layer and an increase in granulation tissue, as well as a decrease in the number of macrophages and neutrophils in the experimental group at the end of the 2nd week. However, at the end of the 3rd week, the stratum keratinosum was formed, the number of fibroblasts and macrophages increased, and the number of neutrophils decreased. At the end of the 4th week, the epidermis was fully developed.	[52]
*Camelia sinensis* leaves 70% (*v*/*v*) ethanolic extract	Wistar albino rats, male	Second-degree thermal burn—hot metal plate (120 °C), applied for 5 s	Once a day (1 g per wound) for 21 days E: 0.6% of extract in Vaseline C: Vaseline NC: normal saline	The average healing time was significantly shorter in the experimental group compared to the control group. In histopathological analysis, a significantly lower number of inflammatory cells was observed in the experimental group during the entire study, and no significant differences in epidermal regeneration and angiogenesis were observed. On the 21st day, angiogenesis was significantly higher, and there were no significant differences in other parameters.	[53]
*Camelia sinensis* leaves 70% (*v*/*v*) ethanolic extract	Wistar albino rats, male	Second-degree thermal burn—metal cube heated to 100 °C, applied for 15 s	Once a day for 14 days E: 2% of extract in normal saline S: 1% SSD cream C: normal saline	The average burn area was significantly smaller in the experimental group compared to the negative control group. No significant differences in burn size, vascularisation, number of inflammatory cells, and epithelisation were observed between the experimental, standard, and control groups.	[54]
*Carissa spinarum* roots methanolic extract	Swiss albino mice, either sex	Second-degree thermal burn—hot aluminium plate (85 °C), applied for 5 s	Once a day for 36 days E: 1 or 2.5% of extract in an ointment base S: 1% SSD cream C: ointment base NC: w/o treatment	From day 20, the experimental and standard groups observed a significant increase in wound closure. Reepithelialisation time was significantly reduced compared to controls in the group treated with 2.5% extract and 1% SSD cream. In the histological examination in the group treated with 2.5% of the extract compared to the other groups, healing was advanced. There was a complete renewal of the epidermis, an increase in the amount of collagen and a decrease in the number of inflammatory cells. In addition, the hydroxyproline content was significantly higher.	[55]
*Centella asiatica* herb 70% (*v*/*v*) ethanolic extract	albino mice, male	Chemical burn—50% phenol solution, applied for 30 s	Once a day for 10 days E: 2% of extract in base gel S: Bioplacenton^®^ jelly (neomycin + placenta extract) C: base gel	Treatment with *C. asiatica* gel improves wound healing compared to the control group but less than in the standard group. Complete wound closure in the experimental group was observed after 8 days, in the standard group after 6 days, while in the control group, after 10 days, the wound closure was only 75.34 ± 20.709%.	[56]
*Centella asiatica* aerial parts n-hexane, ethyl acetate, methanolic and aqueous extracts	Sprague-Dawley rats, male	Second-degree thermal burn—hot plate heated to 75 °C, applied for 10 s	Once a day (0.5 mL per wound) for 14 days E: 10% of appropriate extract in vehicle C: vehicle or normal saline NC: w/o treatment	The degree of wound healing was statistically significantly higher in the experimental groups than in the control groups. After 14 days, it was 53.87 ± 4.64, 57.53 ± 5.68, 60.31 ± 5.70, and 59.82 ± 8.31% for the hexane, ethyl acetate, methanolic, and aqueous extracts, respectively, and 38.07 ± 5.15, 31.85 ± 2.66, and 25.36 ± 1.81% for the vehicle, normal saline, and untreated controls, respectively. In the histopathological analysis of the experimental groups, in contrast to the control groups, fully developed epithelisation and keratinisation were observed without necrosis and inflammation.	[57]
*Cleistocalyx operculatus* leaves hydrodistillation	Swiss albino mice, male	Second-degree thermal burn—aluminium bar heated to 100 °C, applied for 15 s	Once a day (50 µL per wound) for 20 days E: 1% of essential oil (in 0.1% DMSO and Tween 20 solution) S: Tamanu oil C: normal saline	The wound area of the experimental group after 10 and 20 days was significantly smaller than in the control and standard groups. In the histopathological analysis in the experimental group, in contrast to the other groups, re-epithelialisation was completed. Fewer inflammatory cells, thick, neatly arranged fibres, and mature hair follicles were observed.	[58]
*Copaifera officinalis* oleoresin	Swiss mice, male	UVB radiation-induced paw burn model (0.61 mW/cm^2^, 0.75 J/cm^2^)	Once a day (15 mg per paw) for 6 days E: 3% of oleoresin in base cream S: 1% SSD cream C: base cream NC: w/o treatment	Mechanical allodynia lasting 6 days in the untreated group was significantly reduced from the second day of treatment in the experimental group and the third day in the standard group. Irradiation-induced thermal hyperalgesia was abolished more strongly in the experimental group than in the other groups. Infiltration of inflammatory cells after irradiation was significantly reduced in the experimental and standard groups, while increased skin thickness was significantly reduced only in the standard group.	[59]
*Crocus sativus* stigmas 70% (*v*/*v*) ethanolic extract	Sprague-Dawley rats, male	Third-degree thermal burn—aluminium bar boiled in water for 30 s, applied for 10 s	Once a day for 28 days E: 20% of extract in 1% SSD cream S: 1% SSD cream NC: w/o treatment	The percentage of wound closure was significantly higher in the experimental group after 7 days compared to the others. After 14 days, the wounds of the experimental and the standard groups were almost completely closed. In the experimental group, inflammation and redness were significantly reduced, and scarring was minimal. Histopathological analysis after 14 days showed an increase in the number of cells, blood vessels, fibroblasts, and fibrocytes in the experimental group compared to the standard, as well as a comparable, lower than in control, number of inflammatory cells. After 28 days, the number of inflammatory cells, fibrocytes, fibroblasts and total cells significantly decreased in the experimental group compared to the control. In addition, the secretion of interleukin 1β and tumour growth factor β1 was reduced, and the hydroxyproline content was increased.	[60]
*Cucurbita moschata* fruit peel 70% (*v*/*v*) ethanolic extract	Wistar albino rats, male	Second-degree thermal burn—electrical heater (110 °C), applied for 10 s	Once a day for 14 days E: 10 or 20% of extract in Eucerin S: 1% SSD cream C: Eucerin	The degree of wound closure after 14 days was 57.80 ± 5.71, 78.80 ± 3.96, 77.60 ± 5.41, and 90.80 ± 5.86% for the group, standard, treated with 10% extract and treated with 20% extract groups, respectively. It was significantly lowest in the control group and significantly highest in the group treated with 20% of the extract. Tissue analysis of oxidative stress biomarkers showed that lipid peroxidation in the standard and experimental groups was significantly reduced compared to the control group. The total antioxidant power and total thiol molecules content were significantly higher in the standard group and the group treated with 20% extract than in the control group. In histopathological analysis, the group treated with 20% extract showed better signs of wound healing. Inflammatory cells were absent, collagen fibres were well organised, and the basal epithelial layer reached a normal level.	[61]
*Cucurbita moschata* oil	BALB/c albino mice, male	Third-degree thermal burn—coin heated for 3 min with a spirit lamp, applied for 8 s	Once a day for 28 days E: 30 or 40% of oil NC: w/o treatment	Compared to the negative control, the group treated with sesame oil showed better wound healing, higher total antioxidant power, and lower malondialdehyde levels than the control.	[62]
*Ephedra alata* whole plant 2-step extraction with n-hexane and 50% (*v*/*v*) ethanol	Syrian hamsters (*Mesocricetus auratus*), male	Third-degree thermal burn—metal plate boiled in water for 5 min	Once a day for 15 days E: 1.5% of extract in an ointment base C: ointment base NC: w/o treatment	In the macroscopic assessment, the burn wound of the experimental group healed faster and better than in the other groups. However, in the histopathological analysis, no statistically significant differences in the degree of fibrosis and collagen fibres density were observed compared to the control group.	[63]
*Globularia alypum* leaves methanolic extract	Wistar albino rats, male	Second-degree thermal burn—electric heater (110 °C), applied for 10 s	Once a day for 16 days E: extract in glycerol (unspecified concentration) S: Cytol Centella^®^ cream (with *Centella asiatica*) C: glycerol NC: normal saline	From day 12, the wound closure of the experimental group was significantly better compared to the untreated group. The hydroxyproline level in the experimental group was significantly higher than in the other groups. In the histopathological analysis after 16 days, the appearance of the epidermis and skin in the experimental group was normal, while in the standard group, there were signs of inflammation. In the untreated groups, there was a massive infiltration of inflammatory cells without a developed epidermal layer.	[64]
*Glycyrrhiza glabra* roots 75% (*v*/*v*) ethanolic extract	Sprague-Dawley rats, male	Third-degree thermal burn—hot plate	Once a day for 29 days E: 10% of extract in base gel S: 1% SSD cream C: base gel NC: w/o treatment	After 14 days, no signs of wound closure were observed macroscopically in the experimental group. Histopathological evaluation of this group showed complete re-epithelialisation, minimal granulation tissue formation, mild inflammation, and irregular collagen distribution. In contrast, in the standard group, only granulation tissue and severe inflammation were present. Coagulative necrosis of the epidermis without granulation tissue was noted in the untreated groups. After 28 days, there were no differences between the groups in tensile strength, maximum stress, yield strength and stiffness.	[65]
*Gundelia tournefortii* aerial parts unspecified extract	Wistar albino rats, male	Second-degree thermal burn—metal plate heated in a flame for 5 min, applied for 8 s	Once a day for 21 days E: extract with milk-cream (4:1) S: 1% SSD cream NC: w/o treatment	From day 7, the wound dimensions of the experimental group and the standard were significantly smaller than those of the controls. From day 14, significantly greater wound closure was observed in the experimental group than in the other groups. After 21 days, the histopathological analysis in the experimental group showed a reduction in inflammation and an increase in the degree of re-epithelialisation and the length of blood vessels compared to the standard group. In addition, there was a significantly higher total volume of collagen fibres, and the scab covered a larger wound area than in the control group.	[66]
*Hippophaë rhamnoides* leaves aqueous extract	Sprague-Dawley rats, male	Third-degree thermal burn—metal rod heated to 85 °C, applied for 20 s	Twice a day for 7 days E: 2.5, 5, 7.5 or 10% of extract in soft white petroleum S: 1% SSD cream C: soft white petroleum	The wound area on days 4 and 8 was the smallest in the group treated with 5% extract. Also in this group, significantly higher contents of hydroxyproline, hexosamine, protein, matrix metalloproteinase 9, vascular endothelial growth factor, type-III collagen, and antioxidants (glutathione, vitamin c, superoxide dismutase, catalase, glutathione S-transferase and malondialdehyde) were found in the granulation tissue than in control. The epidermis thickness in this group was comparable to the standard group and significantly higher than in the control group, and the density of blood vessels was significantly higher than in the other groups.	[67]
*Hippophaë rhamnoides* seeds oil	Merino sheep, female	Third-degree flame burns—applied with a Bunsen gas burner	Every 6 days for 18 days E: 20 mL of oil C: w/o treatment	The re-epithelialisation time was significantly shortened in the treated group, and the degree of epithelialisation was significantly higher than in the control group. There were no statistically significant differences between the groups in the mean peripheral blood flow and the mean content of malondialdehyde and superoxide dismutase in the wound.	[68]
*Hippophaë rhamnoides* leaves aqueous extract	Sprague-Dawley rats, male	Third-degree thermal burn—metal probe heated to 85 °C, applied for 20 s	Twice a day for 7 days E: 2.5% of extract in soft white petroleum S: 1% SSD cream C: soft white petroleum	In the macroscopic evaluation, the experimental group showed the best wound repair and improvement of the peri-wound skin condition. Compared to the control, the wound of the experimental group had a significantly reduced level of reactive oxygen species and the inflammatory response (3-nitrotyrosinase, nitric oxide synthase-2, tumour necrosis factor α, interleukin 1β, interleukin 6 and NF-κB), as well as significantly increased expression of markers responsible for cell proliferation, epithelial migration, angiogenesis, skin hydration, and cytoprotection (proliferating cell nuclear antigen, cytokeratin-14, cluster of differentiation 31, aquaporin 3, hypoxia-inducible factor 1α, glucose-regulated protein 78, and transient receptor potential vanilloid 3). In contrast to the control and standard groups in the treated group, the tissue was characterised by a well-organised orientation with increased mature collagen and rapid epithelial migration towards the wound bed. Increased activity of hexokinase, citrate synthase, glucose-6-phosphate dehydrogenase, mitochondrial enzyme cytochrome c oxidase and an increase in adenosine triphosphate levels were noted, and lower activity of lactate dehydrogenase.	[69]
*Hypericum perforatum* seeds oil	Sprague-Dawley rats, male	Second-degree thermal burn—iron plate heated in boiling water for 5 min, applied for 20 s	Once a day for 20 days E: 2 mL of oil NC: w/o treatment	In the treatment group, re-epithelialisation was complete after 21 days, while in the control group, no epidermal layer was formed. In the histopathological analysis of the experimental group, significantly fewer inflammatory cells and increased angiogenesis were observed than in the control group.	[70]
*Juglans regia* seeds grounded into ointment	Guangxi Bama mini-pigs, female	Third-degree thermal burn—aluminium plate heated in boiling water for 10 min, applied for 45 s. The wounds were left unhealed for 3 weeks.	After 3 weeks, the wound was cleaned and treated twice daily for 28 days. E: walnut ointment S: recombinant human Epidermal Growth Factor C: normal saline	Compared to the control group, the experimental and standard groups showed a significant reduction in the wound area and improved healing from day 7 to day 28. The total wound closure time was 18.44 ± 3.09, 23.56 ± 4.85 and 32.56 ± 5.36 days in the experimental, standard and control groups, respectively. After 28 days, the histopathological analysis showed significantly thinner proliferative and differentiating epidermis layers in the experimental group. The positive staining of P63 (epidermal proliferation marker) and CK10 (epidermal differentiation marker Cytokeratin 10) was stronger in the standard group compared to the others, but in the experimental group, the positive staining of P63 was stronger than in control.	[29]
*Linum usitatissimum* seeds oil	New Zealand rabbits, male	Second-degree thermal burn—stainless steel cylinder heated in boiling water for 3 min, applied for 15 s	Once a day (1 g per wound) for 28 days E: linseed oil S: Cicatryl-bio^®^ ointment (sodium hyaluronate + allantoin) C: Vaseline NC: w/o treatment	The degree of wound closure was significantly higher in the experimental and standard groups from day 16 than in the other groups. The total wound healing time was 26 ± 5.8, 32.5 ± 2.8, 35.6 ± 3.9 and 35 ± 1.1 days for the experimental, standard, control, and negative control group, respectively. In the histopathological analysis of the experimental group, compared to the other groups, a smaller number of inflammatory cells, complete re-epithelialisation, reduced thickness and fibrosis of the epidermis, and an increase in the number of capillaries, collagen fibres, fibroblasts, and myofibroblasts were observed.	[71]
*Lobelia alsinoides* whole plant ethanolic extract	Wistar albino rats, male	Third-degree thermal burn—metal plate, heated red hot, applied for 30 s	Once a day for 16 days E: 5% or 10% of extract in a simple ointment S: 1% SSD cream C: simple ointment NC: w/o treatment	In the group treated with 10% of the extract, complete macroscopic re-epithelialisation was visible after 12 days, and in the group treated with 5% of the extract after 16 days. In the other groups, after 16 days, the wound was still open and red. In the histopathological analysis after 16 days, the experimental groups, compared to the others, showed neovascularisation, complete re-epithelialisation, fibroblast proliferation, neutrophil infiltration, angiogenesis, increased amount of collagen and decreased inflammation.	[72]
*Malva sylvestris* flowers 70% (*v*/*v*) ethanolic extract	Albino rats, male	Second-degree thermal burn—metal plate heated in boiling water for 5 min, applied for 10 s	Once a day for 35 days E: 5% or 10% of extract in base cream S: 1% SSD cream C: base cream NC: normal saline	There was a significant increase in the percentage of wound closure in the experimental groups from the 7th day. After 8 days, in the experimental groups, an increase in the thickness of the epidermis and granulation tissue was observed, and the organisation of squamous cell maturation and orthokeratin improved. In addition, after 21 days, a higher degree of scar formation, collagen organisation, formation of hair follicles and lymphatic vessels, and the degree of innervation was observed in the experimental groups.	[73]
*Michelia champaca* flowers ethanolic extract	Wistar albino rats, male	Second-degree thermal burn—hot molten wax (80 °C), applied for 8 min	Once a day until healed E: 10% of extract in an ointment base S: 1% SSD cream	The epithelisation period in the experimental group was 18.33 ± 2.42 days and was shorter than in the standard group, which was 21.67 ± 3.01 days.	[74]
*Musa paradisiaca* stems methanolic extract	Wistar albino rats	Third-degree thermal burn—red hot steel rod	Once a day for 14 days E: methanolic extract (unspecified concentration and preparation) C: Vaseline	Compared to the control group, the experimental group showed an increase in wound closure percentage, epithelisation, and faster tissue regeneration.	[75]
*Myrtus communis* leaves ethanolic extract	Wistar albino rats	Third-degree thermal burn—exposed to 90 °C water bath for 10 s	Twice a day for 48 h E: 5% (*w*/*w*) of extract in simple ointment (0.5 g) NC: w/o treatment	In the skin affected by a burn injury, an increase in superoxide dismutase and catalase activity was observed, as was an increase in malondialdehyde and a decrease in glutathione and nitric oxide levels. After the topical application of 5% ointment, a significant reduction in malondialdehyde level, an increase in nitric oxide level, and an increase in superoxide dismutase and catalase were observed. There was no effect on glutathione level and total tissue protein.	[76]
*Nigella sativa* seeds oil	Wistar albino rats, male	Second-degree thermal burn—brass probe heated in boiling water, applied for 20 s	Twice a day for 14 days E: 50% of oil + 50% of cold cream S: 1% SSD cream C: cold cream	The experimental and standard groups observed a reduction in the clinical signs of inflammation, such as warmth, redness, and swelling. The histopathological analysis showed a significant increase in granulation tissue thickness in these groups. After 14 days, the wound appearance of the experimental group was the most normal.	[77]
*Olea europaea* seeds oil	domestic pigs, female	Second-degree thermal burn—aluminium bar preheated to 400 °C, applied for 20 s, the necrotic epidermis was removed using bromelain-derived agent, Debridase^®^	Once a day for 14 days E: purified olive oil S: 1% SSD cream NC: w/o treatment	After 14 days, it was observed that treatment efficiency was significantly better in the standard group. There were no significant differences in wound healing between the experimental and no-treatment groups.	[78]
*Olea europaea* leaves 70% (*v*/*v*) ethanolic extract	Wistar albino rats, male	Third-degree thermal burn—metal plate, heated in 94 °C water for 20 min, applied for 30 s	Twice a day for 21 days E: 10% of extract in Eucerin S: 1% SSD cream NC: w/o treatment	In the macroscopic evaluation from day 14, it was observed that the treated groups had significantly less wound area than the untreated group. Moreover, the wound area of the experimental group was significantly smaller than that of the standard group. In the histopathological evaluation after 14 days, it was observed that the number of neutrophils and macrophages in the wound significantly decreased in the treatment groups, and the number of fibroblasts increased. There were no differences between the groups in the number of endothelial cells.	[79]
*Onosma dichroanthum* roots acetone extract	Wistar albino rats, female	Second-degree thermal burn—metal rod heated in boiling water to 95 °C, applied for 10 s	Once a day for 14 days E: 2% of extract in a base ointment S: 1% SSD cream C: base ointment NC: w/o treatment	After 14 days in the standard group, 2 rats had the wound completely healed, and in the remaining rats, the area was significantly reduced compared to the control groups. On the other hand, in the experimental group, the surface area not only did not decrease but increased its surface area.	[80]
*Onosma bulbotrichum* roots n-hexane and dichloromethane (1:1) extract	rabbits, either sex	Second-degree thermal burn—steel plate heated to 150 °C, applied for 20 s	Twice a day until healed E: 1, 2 or 5% of extract in cold cream S: 1% SSD cream C: cold cream NC: w/o treatment	In the study, it was observed that in the standard group and the group treated with 5% of the extract, the time to complete wound healing was significantly shortened to 16 and 17 days, respectively. In contrast, in the control group and the negative control, it was 24 and 26 days, respectively. Compared to the control groups, the wounds of the treatment groups had a significantly higher content of collagen and non-collagen proteins. The histopathological analysis observed the best wound healing in the group treated with the ointment with 5% extract.	[81]
*Phyllanthus niruri* whole plant ethanolic extract	Wistar albino rats, male	Second-degree thermal burn—hot wax (80 °C), applied for 8 min	Once a day until healed E: 10% of extract in a simple ointment S: 1% SSD cream	Compared to the group treated with 1% silver sulfadiazine cream, topical administration of an emulgel containing 10% of the extract does not reduce the wound area or shorten the epithelisation period.	[82]
*Pistacia atlantica* resin hydrodistillation	Wistar albino rats, female	Third-degree thermal burn—aluminium plate heated to 100 °C, applied for 10 s	Once a day for 14 days E: 5, 10 or 20% of resin oil in the ointment base C: ointment base	There were no significant differences in the size of the wound in the study groups in the macroscopic assessment. In the microscopic and histopathological evaluation, the groups treated with the emulgel with 5 or 10% of the extract developed more capillaries, and significantly higher concentrations of basic fibroblast growth factor and platelet-derived growth factor were observed than in the control group.	[83]
*Pistacia atlantica* mastic gum ethanolic extract and essential oil	albino rabbits, male	Second-degree thermal burn—metal plate	Once a day for 21 days E: 30% (9.12 mL of extract + 24.15 mL of essential oil) or 60% (18.24 mL of extract + 48.3 mL of essential oil) of composition in Eucerin C: 30 or 60% of distilled water in Eucerin	After 21 days, the degree of wound closure was significantly higher and amounted to 65% and 94%, respectively, for the group treated with 30% and 60% of the extract, and for the corresponding control groups, 8% and 10%, respectively. Blood concentrations of glutathione peroxidase, superoxide dismutase, catalase, and HDL in the treatment groups were significantly elevated compared to controls. Contrary to control groups, glucose concentration was not elevated. However, there were no significant differences between the groups in malondialdehyde and LDL concentrations.	[84]
*Pistacia atlantica* resin oil purchased	Sprague-Dawley rats, male	Third-degree thermal burn—aluminium plate heated to 100 °C, applied for 15–20 s	Once a day (200 mg/kg body weight) for 14 days E: resin oil S: 1% SSD cream NC: w/o treatment	The degree of wound closure in the experimental group after 14 days was 98.6 ± 2.5% and was comparable to the standard group (94.7 ± 4.1%) and significantly higher than in the negative control group (71.2 ± 3.4%). The level of superoxide dismutase, glutathione peroxidase, total antioxidant status, vascular endothelial growth factor, and hydroxyproline was significantly higher in the experimental group compared to the negative control group and the standard group. The malondialdehyde level was comparable to the standard group and higher than the control group.	[85]
*Pistacia lentiscus* oil cold pressed	New Zealand rabbits, male	Third-degree thermal burn—metal cylinder heated for 3 min in boiling water, applied for 15 s	Once a day until healed E: 1 mL of oil S: 1% Madecassol^®^ (with *Centella asiatica*) C: Vaseline NC: w/o treatment	The period of complete re-epithelisation in the treatment groups was 30 ± 3.94 and 33.5 ± 3.78 days in the experimental and standard groups, respectively. It was significantly shorter than in the negative control group, which was 37.16 ± 3.54 days, but it was not considerably different from the group receiving Vaseline (34.66 ± 3.88 days).	[86]
*Plantago major* seeds aqueous extract	Sprague-Dawley rats, male	Third-degree thermal burn—hot metal plate	Once a day for 21 days E: 20 or 50% of extract in Eucerin S: 1% SSD cream C: Eucerin	There were no statistically significant differences between the groups in wound size. However, in the histopathological analysis of the wound in the experimental group, in contrast to the control group, good re-epithelialisation and organisation of the granulation tissue were observed. Parallel-oriented fibroblasts were present in the well-structured layer of the epidermis, and the number of inflammatory cells and capillaries was high.	[87]
*Pothos scandens* leaves ethanolic extract	Wistar albino rats, either sex	Thermal burn—iron plate heated in a flame to red hot, applied for 10 s	Once a day for 20 days E: 2, 4, 6, 8 or 10% of extract in glycerol C: glycerol NC: w/o treatment	The extract’s application significantly reduced the re-epithelialisation time compared to the control groups. The shortest time was obtained in the group treated with 4% extract (22 ± 2.43 days). In the control group, the time was 35 ± 1.69 days, and in the negative control group, 40 ± 1.06 days.	[88]
*Punica granatum* peel standardised pomegranate rind extract (13% of ellagic acid)	Wistar albino rats, male	Third-degree thermal burn—metal rod heated to 100 °C in boiling water, applied for 20 s	Once a day (0.5 g per wound) for 12 days E: 1, 2.5 or 5% of extract in formulation base S: 1% SSD cream C: formulation base	Significant differences in the percentage of wound closure between the treatment and control groups were observed from day 4 onwards. The extract reduced the wound surface area in a concentration-dependent manner and was comparable to 1% silver sulfadiazine cream. In addition, in the standard group and, depending on the concentration, in the experimental groups, a significant reduction in myeloperoxidase activity was observed, which is an indicator of inflammatory neutrophil infiltration.	[89]
*Punica granatum* fruits standardised pomegranate extract (40% of ellagic acid)	albino rats (*Rattus norvegicus*), male	Second-degree thermal burn—steel plate heated to 85 °C, applied for 5 s	Twice a day for 14 day E: 2.5, 5 or 10% of extract in a cream base S: 1% SSD cream C: cream base	The experimental groups showed higher re-epithelialisation and collagen levels and reduced neutrophil infiltration and angiogenesis than the control and standard groups.	[90]
*Punica granatum* fruits methanolic extract	minipigs, either sex	Second-degree thermal burn—fuel smeared on the skin and lit by an open fire for 45 s	Twice a day for 28 days E: 5% of extract in base gel S: 1% SSD cream or 20 g/kg Jing Wan Hong herbal ointment C: base gel NC: w/o treatment	After 28 days of treatment, the skin morphology in the experimental group improved and was close to normal skin. Compared to the control, comparable acceleration of healing, shortening of healing time, and elimination of scab and fur growth were observed in the experimental and standard groups. From day 7, the treatment groups showed an increase in vascular endothelial growth factor A and transforming growth factor β1 levels compared to the control groups.	[91]
*Rubus caesius* leaves 70% (*v*/*v*) ethanolic extract	Wistar albino rats, male	Third-degree thermal burn—steel device heated in boiling water (100 °C), applied for 5 s	Once a day for 21 days E: 10% of extract in cold cream S: 1% SSD cream C: cold cream	After 7 days, compared to the control and standard group, a higher number of capillaries was observed in the experimental group, and their number decreased in the following days. After 21 days, an almost complete wound healing with well-rebuilt granulation tissue and no inflammatory cells were observed in the experimental group and incomplete epithelisation and infiltration of inflammatory cells in the control and standard groups.	[92]
*Sambucus nigra* flowers, leaves 70% (*v*/*v*) ethanolic extract	Wistar albino rats, male	Third-degree thermal burn—steel device heated in boiling water (100 °C), applied for 5 s	Once a day for 21 days E: 10% of extract of flowers or leaves in cold cream S: 1% SSD cream C: cold cream	Compared to the control group and the standard, the experimental group had a higher number of capillaries after 7 days, and their number decreased in the following days. After 21 days, an almost complete wound healing with well-rebuilt granulation tissue and no inflammatory cells were observed in the experimental group and incomplete epithelisation and infiltration of inflammatory cells in the control and standard groups.	[92]
*Sauromattum guttatum* tubers 70% (*v*/*v*) methanolic extract	BALB/c mice, either sex	Second-degree thermal burn—metal bar heated on opened flame, applied for 9 s	Three times a day for 15 days E: 2% of extract in petroleum jelly S: 1% SSD cream C: petroleum jelly NC: w/o treatment	After 15 days, the experimental and standard groups showed a reduction in wound area compared to the control group. In the histopathological analysis, in the treated groups, in contrast to the control groups, a normal healing process was observed, i.e., normal regeneration of the epidermis, the presence of new capillaries, granulation tissue, sebaceous glands and hair follicles. In addition, the treatment groups increased the expression of platelet-derived growth factor, epidermal and fibroblast growth factor.	[93]
*Sanguisorba officinalis* roots 70% (*v*/*v*) ethanolic extract	Sprague-Dawley rats, male	Second-degree thermal burn—electrical scald instrument (75 °C), applied for 15 s	Twice a day for 14 days E: 1 mL of extract (100 mg/mL) S: 0.3 g of 1% SSD cream NC: w/o treatment	After 14 days, the experimental and standard groups’ wounds was significantly smaller than the wound of the control group. In the histopathological analysis in the treatment groups, rapid progression of reepithelialisation, formation of granulation tissue, collagen fibres and blood vessels, and disappearance of inflammatory cells were observed in contrast to the negative control.	[94]
*Senna podocarpa* leaves 50% (*v*/*v*) ethanolic extract	Wistar albino mice, either sex	Second-degree chemical burn—hydrochloric acid (0.2 mL, 37%), applied for 15 s	Once a day for 14 days E: 2.5 or 7.5% of extract in base emulgel or extract poultice S: 1% SSD cream C: base emulgel NC: w/o treatment	The degree of wound closure after 14 days was 64, 87, 50 and 66% for the group treated with 2.5% extract, 7.5% extract, extract poultice and 1% SSD cream, respectively. In contrast, in the control groups, the degree of wound closure was below 10%. The group treated with 7.5% extract in emulgel had the best results in histopathological analysis, with significantly higher levels of keratin, epidermal cells, gland cells, adipocytes, and collagen than in the control and standard groups. However, more inflammatory cells and fewer elastin fibres were also observed.	[95]
*Sesamum indicum* oil cold pressed	BALB/c albino mice, male	Third-degree thermal burn—coin heated for 3 min with a spirit lamp, applied for 8 s	Once a day for 28 days E: 30 or 40% of oil NC: w/o treatment	Compared to the negative control, the group treated with sesame oil showed better wound healing. The level of total antioxidant power was significantly higher, and the level of malondialdehyde significantly lower than in control.	[62]
*Terminalia chebula* fruits 70% (*v*/*v*) ethanolic extract	Wistar albino rats, male	Second-degree thermal burn—hot water (90 °C), applied for 6 s	Once a day for 30 days E: 5 or 10% of extract in base cream S: 1% SSD cream C: base cream NC: normal saline	The wound size decreased the most in the group treated with the cream with 10% extract. Significant differences in wound size between this group and the other groups were evident from day 10. Complete wound closure was seen after 20 days in the 10% extract cream group, 25 days in the 5% extract cream group, and 33–35 days in the standard and control groups. In the morphological and histopathological analysis, inflammatory cell infiltration, neovascularisation, fibroblast proliferation, mucopolysaccharide deposition in the matrix, degree of inflammation, the extent of bacterial colonisation, and degree of granulation tissue formation were scored. Statistically, the best results, indicating an advanced degree of healing, were obtained for the group treated with cream with 10% of the extract. The group treated with the cream with 5% extract obtained as good results as the standard group.	[96]
*Tragopogon graminifolius* aerial parts 80% (*v*/*v*) ethanolic extract	Wistar albino rats, male	Second-degree thermal burn—aluminium rod, heated to 110 °C, applied for 10 s	Once a day for 14 days E: 5 or 10% of extract in Eucerin S: 1% SSD cream C: Eucerin	After 14 days, the wound area was reduced by 80, 73, 78, and 58% in the 10% extract, 5% extract, standard, and control groups, respectively. Significant differences were observed in the standard group and the group treated with 10% extract compared to the control. In these two groups, also in the histopathological analysis, re-epithelialisation of the epidermis and a significantly better profile of biomarkers of tissue oxidative stress, including a higher content of total thiol molecules and lower lipid peroxidation, were visible. The control and experimental groups observed no differences in total antioxidant power.	[97]
*Tridax procumbens* leaves 90% (*v/v*) ethanolic extract	Wistar albino rats, male	Second-degree thermal burn—hot wax (80 °C), applied until solidified	Once a day (0.5 g per wound) for 15 days E: 1% of extract in base gel S: 1% SSD cream C: base gel	Wound closure after 15 days was 69.14 ± 0.7497% in the experimental group, 90.43 ± 0.7691 % in the standard group, and 68.58 ± 0.7791% in the control group. The mean re-epithelisation time was 32.10 ± 0.86 days in the experimental group, 25.20 ± 2.10 days in the standard group and 38.36 ± 1.77 days in the control group. There were no statistical comparisons.	[50]
*Viola tricolour* flowers 70% (*v*/*v*) ethanolic extract	Wistar albino rats, male	Sunburn—UVB radiation: 0.27 mW/cm^2^, 0.5 J/cm^2^	Once a day for 6 days E: 1, 3 or 10% of extract in base gel S: 1% SSD cream C: base gel	In the groups treated with 3 and 10% of the extract, static and dynamic allodynia and paw oedema were significantly reduced, and the increase in myeloperoxidase activity was inhibited compared to the control, showing effectiveness comparable to that in the standard group.	[98]
*Vitis vinifera* leaves 30% (*v*/*v*) methanolic extract	Wistar albino rats, male	Second-degree thermal burn—metal plate heated in boiling water for 5 min, applied for 10 s	Twice a day for 21 days E: 0.5, 1, 1.5 or 2% of extract in Eucerin S: 1% SSD cream C: Eucerin NC: w/o treatment	Wound healing in the experimental groups was better than in the control groups. Wound healing in the experimental group was worse (for 0.5, 1 and 1.5%) or comparable (for 2%) than in the standard group. The groups treated with 1.5 and 2% of the extract showed better healing parameters than the control groups, such as the number of hair follicles, sebaceous glands, fibroblasts, macrophages, neutrophils, blood vessels, and the thickness of the epidermal layer.	[47]
*Zanthoxylum bungeanum* seeds oil (expeller pressed)	Sprague-Dawley rats, male	Second-degree thermal burn—hot water (100 °C), applied for 12 s	Twice a day for 7 days, then once a day until healed E: 0.5 or 1 mL of oil per wound S: 1% SSD cream NC: w/o treatment	From day 7 of treatment, a dose-dependent increase in wound healing was observed in the experimental groups compared to the controls. The increase for the group treated with 1000 µL of oil was comparable to the standard group. Histopathological analysis showed a statistically significantly thicker epidermal layer in the treated groups compared to the controls, but no significant differences in skin thickness were found. Levels of superoxide dismutase, hydroxyproline, and type-III collagen were significantly higher in experimental and standard groups compared to controls. However, the level of malondialdehyde, matrix metalloproteinase 2, matrix metalloproteinase 9, tumour necrosis factor α, interleukin 6, interleukin 1β, phosphor-nuclear factor-κB p65, and phosphor-inhibitor of nuclear factor-κB subunit α was significantly lower in these groups compared to controls.	[99]

* C—control group; E—experimental groups; NC—negative control group; S—standard/positive control group; SSD— silver sulfadiazine; w/o—without.

**Table 2:** In vivo studies on animal models—a mixture of preparations.

Composition of the Mixture	Animal Model	Burn Wound	Treatment Schedule *	Results	Ref.
*Allium sativum* (bulbs; squeezed juice)Euphorbia honey	Wistar albino rats, either sex	Thermal burn—metal plate, heated in boiling water for 10 min, applied for 20 s	Once a day until healed E: Euphorbia honey or a mixture of Euphorbia honey and *Allium sativum* juice (amount and concentration not given) S: Betadine solution or 1% SSD cream	The shortest time needed for complete epithelisation and wound closure was noted for the group treated with the mixture, 1% SSD cream, and then for the group treated with Euphorbia honey. The longest time was recorded in the group treated with betadine. In the histological examination, the group treated with the mixture was characterised by a thicker layer of epidermis and skin than the other groups. There were no differences between the groups in the interdigitation index and the orientation of collagen fibres.	[100]
*Aloe vera* leaves *Vitis vinifera* leaves 30% (*v*/*v*) methanolic extracts	Wistar albino rats, male	Second-degree thermal burn—metal plate heated in boiling water for 5 min, applied for 10 s	Twice a day for 21 days E: the combination of leaf extracts in a ratio of 1.5%:1.5% in Eucerin S: 1% SSD cream C: Eucerin NC: w/o treatment	The wound area of the experimental group after 7, 14 and 21 days was significantly smaller than the wounds of the control groups but comparable to the standard group. It has been observed that treatment with the composition has better healing effects than treatment with the individual components. Compared to the control and standard groups, the experimental group showed a higher degree of tissue maturation and organisation and re-epithelialisation, a fully formed epidermis, more hair follicles, sebaceous glands, fibroblasts and capillaries, and a decrease in neutrophil and macrophage infiltration.	[47]
*Arctocarpus heterophyllus,* fruits *Murraya koenigii* leaves *Nerium indicum,* leaves *Punica granatum,* bark 70% (*v*/*v*) ethanolic extracts	albino rats, either sex	Third-degree chemical burn—sulphuric acid, applied for 10 s	Once a day until healed E: 10% or 15% of the combination of extracts (1:1:1:1) in ointment base or base gel S: Povidone-iodine C: ointment base or base gel NC: w/o treatment	The period of epithelialisation in the experimental groups with the basic ointment, the basic gel, and the standard group was significantly shorter than in the corresponding control groups. In addition, it was shorter than in the groups treated with single components. Hydroxyproline level and tensile strength were higher in the experimental and standard groups than in the controls. In the histopathological analysis, better wound healing parameters were observed in the experimental groups with base ointment than with base gel.	[101]
*Azadirachta indica* leaves *Tridax procumbens* leaves Honey 90% (*v*/*v*) ethanolic leaf extracts	Wistar albino rats, male	Second-degree thermal burn—hot molten wax (80 °C), applied until solidified	Once a day for 15 days E: herbal gel (unspecified concentration) S: 1% SSD cream C: base gel	The degree of wound closure after 15 days was 89.35 ± 0.4155, 90.43 ± 0.7691 and 68.58 ± 0.7791%, and the period of epithelisation was 26.32 ± 2.22, 25.20 ± 2.10 and 38.36 ± 1.77 days in the experimental, standard and control groups, respectively. The results obtained in the experimental group were as good as in the standard group and significantly better than in the control group. Moreover, the formulation has been shown to have synergistic activity in wound healing as the results obtained are better than those of the single components of the formulation.	[50]
*Calendula officinalis* *Rosa damascena* Beeswax	Wistar albino rats, male	Second and third-degree thermal burn—metal plate heated in boiling water for 5 min, applied for 10 or 30 s	Once a day for 40 days E: commercial herbal ointment Robacin^®^ (the exact composition is not given) S: 1% SSD cream or *Aloe vera* cream	In the second-degree burn group treated with herbal ointment, wound healing was significantly fastest for the first two weeks and comparable to the standard groups. In the third-degree burn group, wound closure was the fastest in the experimental group. In both burns (second and third degree), a much smaller extent of angiogenesis and fibrosis was observed in the experimental group than in the standard groups. In third-degree burns, epithelialisation in the experimental group was as good as in the *Aloe vera* cream treatment.	[102]
*Cannabis sativa* *Juglans regia* *Pistacia atlantica* *Sesamum indicum* cold pressed oils	albino mice, male	Third-degree thermal burn—boiling water (100 °C), applied for 10 s	Twice a day for 21 days E: the combination of sesame oil (60%), pistachio oil (20%), hemp oil (12%) and walnut oil (8%) S: 1% SSD cream NC: w/o treatment	The degree of wound closure was 99.5 ± 0.8, 78.0 ± 4.0 and 88.4 ± 2.5% in the experimental, standard and control groups. The differences between the groups were statistically significant. In the experimental, standard and control groups, the time to complete epithelisation was 20.5 ± 1.37, 26.33 ± 0.81 and 25.5 ± 0.83 days. It was significantly shorter in the group treated with the oil composition.	[103]
*Centella asiatica,* herb, 70% (*v/v*) ethanolic extract Papaya latex, dried and powdered	albino mice, male	Chemical burn—50% phenol solution, applied for 30 s	Once a day for 10 days E: *C. asiatica* and papaya latex in a ratio of 1%:1%, 0.5%:1.5% and 1.5%:0.5% in a base gel S: Bioplacenton^®^ jelly (neomycin + placenta extract) C: base gel	Complete wound closure was achieved after 6 days in the experimental group in the ratio of 1:1 and in the standard group. After 7 days, it was achieved in the groups in the ratio of 1.5:0.5 and 0.5:1.5. After 10 days, in the control group, the wound closure was only 75.34 ± 20.709%. The combination of *C. asiatica* and papaya latex showed a synergistic effect on wound healing, as in the case of the components used alone. Complete wound closure was observed after 8 days.	[56]
*Cucurbita moschata* *Sesamum indicum* oils	BALB/c albino mice, male	Third-degree thermal burn—coin heated for 3 min with a spirit lamp, applied for 8 s	Once a day for 28 days E: the combination of oils (1:1) NC: w/o treatment	In the experimental groups, wound healing was significantly better than in the negative control and the groups treated separately with sesame oil and pumpkin oil. In addition, a significantly higher level of total antioxidant power and a lower level of malondialdehyde were obtained than in the other groups.	[62]
*Malva sylvestris,* leaves, aqueous extract *Rosa damascena,* petal powder, sesame oil extract *Solanum nigrum,* leaves, aqueous extract	Wistar albino rats, male	Second-degree thermal burn—electrical heater heated to 110 °C, applied for 10 s	Once a day for 14 days E: herbal ointment (5% of each aqueous extract and 33% of oily extract in base ointment) S: 1% SSD cream C: base ointment NC: without treatment	After 14 days of treatment, the wound closure was the highest in the group treated with herbal ointment and amounted to 87.0 ± 2.1%. The remaining groups were 70.8 ± 3.5, 57.0 ± 5.3 and 32.2 ± 1.6% in the standard, control, and negative control groups. Compared to the other groups, the histopathological analysis in the experimental group showed a significant improvement in wound healing with complete re-epithelialisation, well-formed granulation tissue and mild infiltration of inflammatory cells. In addition, advanced neovascularisation, and irregular distribution of myofibroblasts, fibroblasts and collagen fibres were present.	[104]
*Momordica charantia,* fruits *Piper nigrum,* fruits *Pongamia glabra,* leaves aqueous extracts	albino rats, either sex	Third-degree chemical burn—sulphuric acid, applied for 10 s	Once a day for 21 days E: 10% or 15% of the combination of extract (1:1:1) in the ointment base S: Povidone-iodine ointment C: ointment base NC: w/o treatment	The period of epithelialisation was 14.97 ± 0.256, 14.77 ± 0.207 and 15.5 ± 0.315 days in the group treated with 10% ointment, 15% ointment and standard, respectively, and was significantly shorter in these groups than in the control groups, where it was 18.18 ± 0.345 and 18.88 ± 0.259 days in the control and negative control groups, respectively. Hydroxyproline levels and tensile strength were significantly higher in experimental and standard groups compared to controls. In the histopathological analysis in the experimental and standard groups, better wound healing, fewer inflammatory cells, a thicker layer of granulation tissue and epidermis, and more dermal fibrosis were observed than in control.	[105]
*Rhododendron macrophyllum* *Thymus serpyllum*	Wistar albino rats, male	Second and third-degree thermal burn—metal plate heated in boiling water for 5 min, applied for 10 or 30 s	Once a day for 40 days E: commercial herbal ointment Rimojen^®^ (the exact composition is not given) S: 1% SSD cream or *Aloe vera* cream	In both the second and third-degree burn groups, wounds treated with herbal ointment healed much more slowly than those treated with *Aloe vera* cream and 1% silver sulfadiazine cream.	[102]
*Sesamum indicum* oil Camphora Honey	Wistar albino rats, male	Second-degree thermal burn—hot metal plate, applied for 10 s	Once a day for 28 days E: herbal ointment (the exact composition is not given) C: Vaseline	The percentage of wound healing from day 7 was higher for the group treated with herbal ointment than Vaseline. Moreover, neovascularisation was higher in the group treated with herbal ointment than in the control group.	[106]

* C—control group; E—experimental groups; NC—negative control group; S—standard/positive control group; SSD—silver sulfadiazine; w/o—without.

## Data Availability

The data presented in this study are available on request from the corresponding author.

## Supplementary Materials

The following supporting information can be downloaded at: https://www.mdpi.com/article/10.3390/pharmaceutics15020633/s1, Table S1: Summary of clinical trials—single preparations; Table S2: Summary of clinical trials—mixtures of preparations.
