# Supplementary material for: The Potential of Medicinal Plants and Natural Products in the Treatment of Burns and Sunburn—A Review
Source: Pharmaceutics. 2023 Feb 13;15(2):633. doi: 10.3390/pharmaceutics15020633 (PMC9958865; doi:10.3390/pharmaceutics15020633)
Supplement: Supplementary file 1 [file pharmaceutics-15-00633-s001.zip › pharmaceutics-2195381-supplementary.pdf]

pharmaceutics

MDPI

SUPPLEMENTARY MATERIALS

The Potential of Medicinal Plants and Natural Products in the Treatment of Burns and Sunburn—A Review

Weronika Skowrońska and Agnieszka Bazylko *

Department of Pharmaceutical Biology, Faculty of Pharmacy, Medical University of Warsaw, Banacha 1, 02-097 Warsaw, Poland; weronika.skowronska@wum.edu.pl
* Correspondence: agnieszka.bazylko@wum.edu.pl; Tel.: +48-225720959

Table S1. Clinical trials – single preparations

Plant material	Patients	Test groups*	The course of the clinical trial	Results compared to the control group	Ref.
Albizia julibrissin stem bark<br><br>60% (*v*/*v*) ethanolic extract	40 patients with second-degree thermal burn	E: 5% (w/w) of extract in base gel<br>C: 1% SSD cream	Dressing changed once a day for 30 days.	After 30 days, pain and inflammation were reduced in the experimental group. There were no significant differences in itching, redness, swelling, purulent discharge, and skin discolouration.	[13]
Aloe vera inner gel<br><br>spray-dried	30 patients with two similar burns	E: 0.5% of powder in a base cream<br>C: 1% SSD cream	Dressing changed twice a day until complete healing.	Using a cream with *Aloe vera* significantly shortened the time to complete wound healing and resulted in a faster reduction of its surface.	[15]
Aloe vera inner gel	50 patients with second-degree burn	E: 98% *Aloe vera* gel<br>C: 1% SSD cream	Dressing changed twice a day until complete healing.	The study showed that using *Aloe vera* gel significantly shortens the re-epithelialization time, alleviates pain symptoms, and is more cost-effective.	[16]
Arnebia euchroma roots<br><br>heated with goat fat, cow butter and glycerin	45 patients with two similar burns	E: 10% *Arnebia euchroma* ointment<br>C: 1% SSD cream	Dressing changed once a day until complete healing.	*Arnebia euchroma* ointment reduces the burn surface, shortens the healing time, reduces the burning sensation and pain by patients, and increases the warming of the wound. Moreover, it was better tolerated by patients and physicians.	[18]
Betula pendula<br>*Betula pubescens* bark<br><br>triterpene extract (standardized for 72-88% of betulin)	57 patients with two similar burns or one major burn divided into two halves	E: Oleogel-S10 (10% of extract in sunflower oil)<br>C: Octenilin®	Dressing changed every 2 days for 21 days.	The study showed that using Oleogel-S10 significantly shortened the time to complete wound closure and increased the percentage of epithelialization. Moreover, it was better tolerated by patients.	[21]
Camelia sinensis leaves<br><br>aqueous extract	50 patients with second-degree thermal burn	E: 10% of extract in a base cream<br>C: 1% SSD cream	Dressing changed once a day until complete healing.	After 14 days, there were no significant differences in the treatment effects between groups.	[23]
Centella asiatica leaves<br><br>butanolic fraction of ethanolic extract	60 patients with second-degree thermal burn	E: Centiderm® ointment (containing 3% of the fraction)<br>C: 1% SSD cream	Dressing changed once a day until complete healing.	After 14 days, all analyzed parameters, i. e. pliability, vascularity, pigmentation, height, scoring according to Visual Acuity Scale and Vancouver Scar Scale, dryness, itching, and irritation, were assessed statistically significantly better. In addition, the mean time to re-epithelialization and complete healing was significantly shorter.	[25]

Hippophaë rhamnoides fruits<br><br>(unspecified preparation)	60 patients with second-degree thermal burn	E: sea buckthorn cream (containing 40% of active ingredient)<br>C: 1% SSD cream	Cream (3 mm thick) was applied once a day until complete healing.	The average wound healing time was statistically significantly lower.	[28]
Juglans regia seeds<br><br>heated and grounded into an ointment	411 patients with non-healing burn wounds	E: walnut ointment<br>C1: wound debridement and skin autograft<br>C2: antimicrobial agent + recombinant human Epidermal Growth Factor	Daily application of a 1-2 mm thick layer to the wound or surgery.	The effectiveness was comparable to the C1 group and was significantly better than in the C2 group. In E and C1 groups, the time to complete wound healing was significantly shorter.	[29]

* E – experimental; C – control; SSD – silver sulfadiazine

Table S2. Clinical trials – mixtures of natural preparations

Composition	Patients	Test groups*	The course of the clinical trial	Results compared to the control group	Ref.
Alkanna tinctoria (unspecified part)<br>Beeswax<br>Olive oil	64 patients with thermal burn	E: dressing saturated with the herbal mixture (3% of beeswax and 5% of *Alkanna tinctoria* in olive oil)<br>C: dressing saturated with nitrofurazone and rifamycin	The dressings were changed every two days until complete healing.	A significant reduction in the time to start re-epithelialization and decreased pain experienced by patients was observed. In addition, the time of their hospitalization was significantly shortened.	[30]
Aloe vera (spray-dried powdered gel)<br>*Centella asiatica* (commercial cream Cosmelene®)	35 patients with second-degree thermal burn	E: herbal dressing (impregnated with lipocolloids, 5% of Cosmelene®, and 2.5% of aloe powder)<br>C: Bactigras® dressing (impregnated with soft paraffin and 0.5% chlorhexidine acetate)	The dressings were changed every three days until complete healing.	The time to complete healing and the time of the patient's stay in the hospital were significantly shortened. Moreover, the average pain experienced by patients was lower.	[31]
Aloe vera gel<br>*Lavandula stoechas* essential oil<br>*Pelargonium roseum* essential oil	111 patients with second-degree thermal burn	E: herbal cream (unspecified composition)<br>C: 1% SSD cream	The dressings were changed every day until complete healing.	Significantly less pain sensation was observed from the 7th day. There was no change in the prevalence of dry skin.	[32]
Azadirachta indica oil<br>*Hypericum perforatum* oil	9 paediatric patients with second and third-degree thermal burn, keloid ulceration and partial failures of skin graft	E: 1 Primary Wound Dressing® spray composed of neem oil and hypericum oil (unspecified proportions)	The dressings were changed every two days until complete healing.	The preparation induces the formation of granulation tissue and epithelialization and relieves pain.	[33]
Lawsonia inermis (unspecified part)<br>Beeswax	50 patients with second-degree thermal burn	E: herbal ointment (unspecified composition)<br>C: 1% SSD cream	The dressings were changed every day until complete healing.	The average wound healing time was statistically shorter in the experimental group.	[34]
Matricaria chamomilla (unspecified part)<br>*Rosa canina* (unspecified part)<br>Beeswax	60 patients with second-degree thermal burn	E: Adibderm® ointment (unspecified composition)<br>C: 1% SSD cream	The dressings were changed every 6 hours until complete healing.	The average wound healing time was statistically shorter in the experimental group.	[35]

* E – experimental; C – control; SSD – silver sulfadiazine
